# Activation of cGAS‐STING by Lethal Malaria N67C Dictates Immunity and Mortality through Induction of CD11b^+^Ly6C^hi^ Proinflammatory Monocytes

**DOI:** 10.1002/advs.202103701

**Published:** 2022-05-29

**Authors:** Yang Du, Yien Luo, Zhiqiang Hu, Jiansen Lu, Xin Liu, Changsheng Xing, Jian Wu, Tianhao Duan, Junjun Chu, Helen Y. Wang, Xin‐zhuan Su, Xiao Yu, Rong‐Fu Wang

**Affiliations:** ^1^ Department of Medicine and Norris Comprehensive Cancer Center Keck School of Medicine University of Southern California Los Angeles CA 90033 USA; ^2^ Department of Immunology Guangdong Provincial Key Lab of Single Cell Technology and Application School of Basic Medical Sciences Southern Medical University Guangzhou Guangdong 510515 China; ^3^ Department of Joint Surgery The Fifth Affiliated Hospital Southern Medical University Guangzhou Guangdong 510515 China; ^4^ Department of Pediatrics Children's Hospital Keck School of Medicine University of Southern California Los Angeles CA 90027 USA; ^5^ Center for Inflammation and Epigenetics Houston Methodist Research Institute Houston TX 77030 USA; ^6^ Department of Neurology Xiangya Hospital Central South University Changsha Hunan 410008 China; ^7^ Malaria Functional Genomics Section Laboratory of Malaria and Vector Research National Institute of Allergy and Infectious Diseases National Institutes of Health Bethesda MD 20892 USA

**Keywords:** Cyclic GMP‐AMP synthase /STING signaling, interleukin‐6, malaria, proinflammatory monocytes

## Abstract

Cyclic GMP‐AMP synthase (cGAS) and stimulator of interferon genes (STING) play critical roles in the innate immunity against infectious diseases and are required to link pathogen DNA sensing to immune responses. However, the mechanisms by which cGAS‐STING‐induced cytokines suppress the adaptive immune response against malaria infections remain poorly understood. Here, cGAS‐STING signaling is identified to play a detrimental role in regulating anti‐malaria immunity. cGAS or STING deficiency in mice markedly prolongs mouse survival during lethal malaria *Plasmodium yoelii nigeriensis* N67C infections by reducing late interleukin (IL)‐6 production. Mechanistically, cGAS/STING recruits myeloid differentiation factor 88 (MyD88) and specifically induces the p38‐dependent signaling pathway for late IL‐6 production, which, in turn, expands CD11b^+^Ly6C^hi^ proinflammatory monocytes to inhibit immunity. Moreover, the blockage or ablation of the cGAS‐STING‐MyD88‐p38‐IL‐6 signaling axis or the depletion of CD11b^+^Ly6C^hi^ proinflammatory monocytes provides mice a significant survival benefit during N67C and other lethal malaria‐strain infections. Taken together, these findings identify a previously unrecognized detrimental role of cGAS‐STING‐MyD88‐p38 axis in infectious diseases through triggering the late IL‐6 production and proinflammatory monocyte expansion and provide insight into how targeting the DNA sensing pathway, dysregulated cytokines, and proinflammatory monocytes enhances immunity against infection.

## Graphical abstract

Cyclic GMP‐AMP synthase (cGAS) and stimulator of interferon genes (STING) play critical roles in innate immunity against infectious diseases, while the mechanisms that cGAS‐STING‐induced cytokines suppress immune response against malaria infection remain poorly understood. Here, this study reveals that cGAS‐STING signaling plays a detrimental role in regulating anti‐malaria immunity by cooperating with MyD88 to induce p38‐dependent late IL‐6 production and expanding CD11b^+^Ly6C^hi^ proinflammatory monocytes.

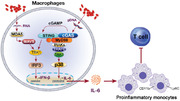

## 1 Introduction

Malaria is a deadly infectious disease that affects 241 million people worldwide; it resulted in about 627 000 global deaths in 2020 [WHO, 2021] due to a lack of effective vaccines against malaria.^[^ ^1^, ^2^, ^3^, ^4^ ^]^ However, there are several obstacles to developing effective vaccines against *Plasmodium*, which include the complexity of the parasite's life cycle and a poor understanding of the parasite's interactions with host immune responses,^[^ ^1^, ^5^, ^6^, ^7^ ^]^ especially the innate immune response, which is key to controlling disease pathogenesis and the severity of malaria infections.^[^ ^5^ ^]^ Furthermore, it is fundamentally important to understand the malaria strain‐specific and strain‐shared innate immune signaling as well as the regulatory mechanisms of innate immune signaling in the cytokine production, disease progression and host mortality of malaria infections.^[^ ^8^, ^9^ ^]^

The innate immune response is activated by recognizing pathogen‐associated molecular patterns (PAMPs)^[^ ^10^ ^]^ through toll‐like receptors (TLRs), RIG‐I‐like receptors (melanoma differentiation‐associated protein 5 [MDA5], retinoic acid‐inducible gene I [RIG‐I] and LGP2), NOD‐like receptors (NLRs), C‐type lectin receptors (CLRs), Absent In Melanoma 2 (AIM2)‐like receptors (ALRs), DNA sensors (cGAMP synthase [cGAS], AIM2, interferon‐gamma induced protein 16 [IFI16], DNA‐dependent activator of IFN‐regulatory factors [DAI], DEAD‐Box Helicase 41 [DDX41], RNA pol III, DNA‐dependent protein kinase [DNA‐PK] and meiotic recombination 11 [MRE11]) and RNA sensors (DEAD/DEAH‐box helicases, heterogeneous nuclear ribonucleoproteins [hnRNPs] and Z‐DNA binding protein 1 [ZBP1]).^[^ ^10^, ^11^, ^12^, ^13^, ^14^, ^15^ ^]^ We and others have demonstrated that TLR7 (but not other TLRs) in specialized plasmacytoid dendritic cells (pDCs) can trigger robust MyD88‐dependent interferon‐regulatory factor 7 (IRF7)‐mediated type‐I interferon (IFN) signaling, producing large amounts of IFN‐α and IFN‐β in response to infections of the lethal strain *Plasmodium yoelii* YM (YM for short).^[^ ^16^, ^17^ ^]^ An early robust production of type‐I IFN (at day one post‐infection [p.i.]) plays a critical role in inducing immunity against blood‐stage lethal malaria YM infections^[^ ^9^, ^16^, ^18^ ^]^ and liver‐stage malaria infections.^[^ ^19^ ^]^ To understand the molecular mechanisms that are responsible for the early robust production of type‐I IFN, we showed that mice deficient in DNA sensor/signaling molecules (cGAS and STING), RNA sensor/signaling molecules (MDA5 and mitochondrial antiviral‐signaling protein [MAVS]) or inflammasome sensor/signaling molecules (NLR family pyrin domain containing 3 [NLRP3], AIM2, Caspase‐1, and IL‐1R) are resistant to lethal malaria YM infections.^[^ ^16^, ^18^ ^]^ All three signaling pathways converge at the IRF3 activation, leading to the upregulation of suppressor of cytokine signaling 1 (SOCS1) expression in wild‐type (WT) pDCs. This upregulation inhibits TLR7‐MyD88‐IRF7‐mediated type‐I IFN signaling and IFN‐α/β production in the early phase (24 h p.i.) of such an infection.^[^ ^16^, ^18^ ^]^ However, IRF7 is not constitutively expressed in other immune cells, such as macrophages, so there is no TLR7‐MyD88‐IRF7‐mediated type‐I IFN signaling pathway in those cells.^[^ ^20^, ^21^ ^]^ Very low levels of IFN‐α/β are produced through only the classical IRF3‐mediated type‐I IFN signaling pathway.^[^ ^16^ ^]^ Moreover, studies have shown that the chronic or late production of type‐I IFN (at day four p.i.) can dampen host immunity against lethal *P*. *berghei* ANKA malaria or vaccine‐induced immunity against *P. y*. 17XNL infections.^[^ ^22^, ^23^, ^24^, ^25^, ^26^ ^]^ Thus, it appears that through innate immune signaling, the timing and magnitude of type‐I IFN dictates the fate (the induction or inhibition) of the anti‐malaria adaptive immune response.

cGAS‐STING signaling is well‐known to detect pathogenic DNA to initiate a strong type‐I IFN response against pathogen infections.^[^ ^27^ ^]^ Emerging evidence has shown that STING is involved in the activation of the nuclear factor‐kappa B (NF‐κB) and mitogen‐activated protein kinase (MAPK) pathways.^[^ ^28^ ^]^ TANK‐binding kinase 1 (TBK1) interacts with STING and contributes to the dsDNA‐mediated activation of NF‐κB.^[^ ^29^, ^30^ ^]^ However, the C‐terminal domain of STING can recruit TNF receptor associated factor 6 (TRAF6) and activate NF‐κB signaling in zebrafish.^[^ ^31^ ^]^ Thus, the complex mechanisms by which cGAS and STING initiate the NF‐κB and MAPK pathways for the production of cytokine (such as IL‐6) remain unclear.

In this study, we report unexpected findings that mice deficient in DNA sensor/signaling molecules (cGAS and STING) are resistant to lethal *P. y. nigeriensis* N67C (N67C for short) malaria infections, and mice deficient in RNA sensor/signaling molecules (MDA5 and MAVS) are sensitive to N67C infections. The activation of cGAS‐STING signaling results in the late production of IL‐6 (at day four p.i. [N67C]), which is closely associated with the sensitive phenotype. Using biochemical and genetic approaches (an anti‐IL‐6R antibody blockade, IL‐6 knockout [KO], MyD88 KO or myeloid‐specific p38 KO mice), we demonstrate that a blockade of the cGAS/STING‐MyD88‐p38‐IL‐6 signaling axis provides mice resistance to lethal N67C infections. Mechanistically, cGAS‐STING activation triggers MyD88‐p38 signaling in macrophages for a late IL‐6 production, which, in turn, induces and expands CD11b^+^Ly6C^hi^ proinflammatory monocytes to inhibit immunity against lethal N67C infections. Indeed, the depletion of CD11b^+^Ly6C^hi^ proinflammatory monocytes could confer a resistance in response to lethal N67C infections. Taken together, these findings provide clarity regarding the previously unrecognized role of the cGAS‐STING‐MyD88‐p38 axis in modulating immunity against lethal malaria infections, which provides potential opportunities for exploring novel therapies.

## 2 Results

### 2.1 The Late Production of IL‐6 is Linked to cGAS‐STING Signaling and is Inversely Correlated with a Resistance to Lethal *P*. *y*. N67C Infections

**Figure 1:**
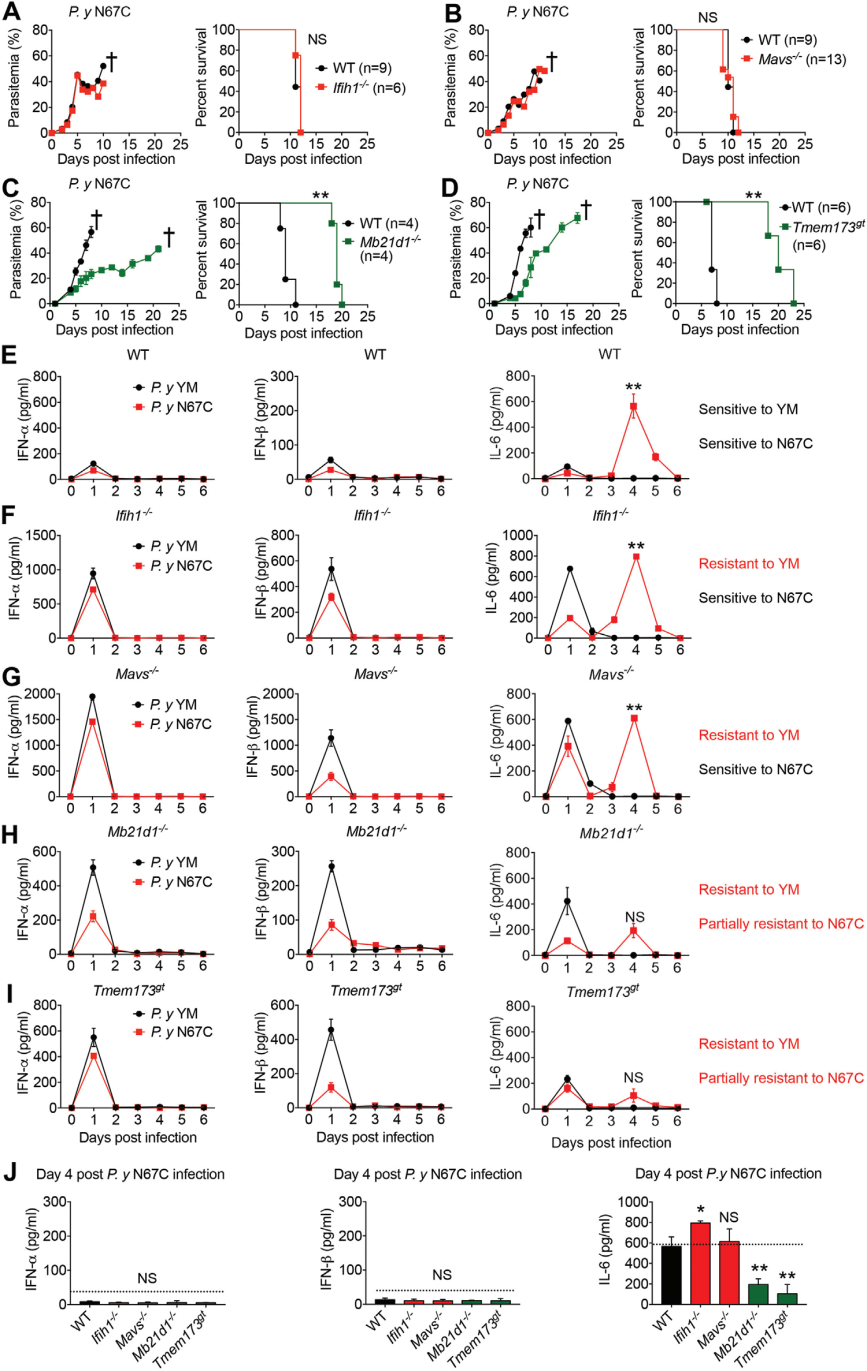
Mice deficient in cGAS/STING produce less late stage IL‐6 and show resistance to lethal *P. yoelii* N67C infection. A–D) WT (black lines), *Ifih1^–/‐^* (A), *Mavs^–/‐^* (B), *Mb21d1^–/‐^* (C), and *Tmem173^gt^* (D) mice were intraperitoneally infected with N67C (1×10^6^ iRBCs). Daily parasitemia and mortality rates are shown. E–I) WT (E), *Ifih1^–/–^* (F), *Mavs^–/–^* (G), *Mb21d1^–/–^* (H) and *Tmem173^gt^* (I) mice (*n* = 3) were intraperitoneally infected with YM (1×10^6^ iRBCs) or N67C (1×10^6^ iRBCs). Sera were collected at indicated time points post infection and subjected to ELISA analysis of IFN‐α, IFN‐β, and IL‐6. J) WT, *Ifih1^–/–^*, *Mavs^–/–^*, *Mb21d1^–/–^* and *Tmem173^gt^* mice (*n* = 5) were intraperitoneally infected with N67C. Sera were collected at day 4 p.i. and subjected to ELISA analysis of IFN‐α, IFN‐β, and IL‐6. Data are representative of three independent experiments and are plotted as the mean ± SD. ^**^ *p* < 0.01 versus corresponding control. NS, not significant. † denotes mouse death.

As we previously mentioned, mice deficient in DNA sensor/signaling molecules (cGAS and STING) and RNA sensor/signaling molecules (MDA5 and MAVS) are resistant to infections of the lethal malaria strain YM through the cross‐regulatory mechanism of the early robust production of IFN‐α/β by two type‐I IFN signaling pathways in pDCs.^[^ ^16^, ^18^ ^]^ We therefore sought to determine whether such a key regulatory mechanism could be applied to other lethal malaria strains. To this end, we infected WT, *Ifih1^–/–^* (coded for MDA5), *Mavs^–/–^*, *Mb21d1^–/–^* (coded for cGAS), and *Tmem173^gt^* (coded for STING) mice, as well as *Tlr7^–/–^* and *Tlr9^–/–^* mice, with lethal doses of N67C (1×10^6^ iRBCs). We unexpectedly found that like the WT mice, the *Mda5^–/–^* and *Mavs^–/–^* mice were sensitive to lethal N67C infections (**Figure** **1**A,B) and died within 10 days p.i. Similarly, the *Tlr7^–/–^* and *Tlr9^–/–^* mice were sensitive to N67C infections and died within 10 days p.i. (Figure S1A,B, Supporting Information). In contrast, the *Mb21d1^–/–^* and *Tmem173^gt^* mice were partially resistant to lethal N67C infections, presented lower parasitemia levels and died at 20 days p.i. (Figure 1C,D).

Since both DNA and RNA sensors trigger IRF3‐mediated type‐I IFN signaling, we considered why the MDA5/MAVS‐deficient, or cGAS/STING‐deficient, mice responded to N67C infections differently. To identify potential cytokine‐production patterns in WT and KO mice in response to N67C or YM infection, we infected WT, *Ifih1^–/–^, Mavs^–/–^*, *Mb21d1^–/–^*, and *Tmem173^gt^* mice with either N67C or YM, then examined the serum amounts of IFN‐α, IFN‐β, and IL‐6 during the course of each infection. We found that little or low serum amounts of IFN‐α, IFN‐β, and IL‐6 were present in the WT mice 24 h after the N67C and YM infections, but the serum amounts of IFN‐α, IFN‐β, and IL‐6 were markedly increased in all the KO mice (Figure 1E–I; Figure S1C, Supporting Information), suggesting that MDA5, MAVS, cGAS, or STING deficiencies promote an early robust cytokine production in response to N67C or YM infections. This was consistent with our previous report^[^ ^16^ ^]^ that MDA5, MAVS, cGAS, or STING could trigger an IRF3‐mediated expression of SOCS1, which suppresses the TLR7‐MyD88‐IRF7‐mediated early production of IFN‐α and IFN‐β. Next, we examined cytokine production in the mice at the late time point. We found no IFN‐α/β or IL‐6 present after day one p.i. (i.e., days two to six p.i.) for the YM infections, regardless of whether the mice were WT or KO (Figure 1E–I). However, the N67C‐sensitive WT, *Mda5^–/–^*, and *Mavs^–/–^* mice produced large amounts of IL‐6 at day four p.i., while the N67C partially resistant *Mb21d1^–/–^* and *Tmem173^gt^* mice produced little or low amounts of IL‐6 at day four p.i. (Figure 1E–J). This suggested that a cGAS‐STING‐induced IL‐6 production at day four may play a key role in dampening the anti‐malaria immunity against lethal malaria N67C infections.

### 2.2 Macrophages are the Main Sources of the IL‐6 Produced at Day Four Post N67C Infection and are Detrimental to Mouse Survival

**Figure 2:**
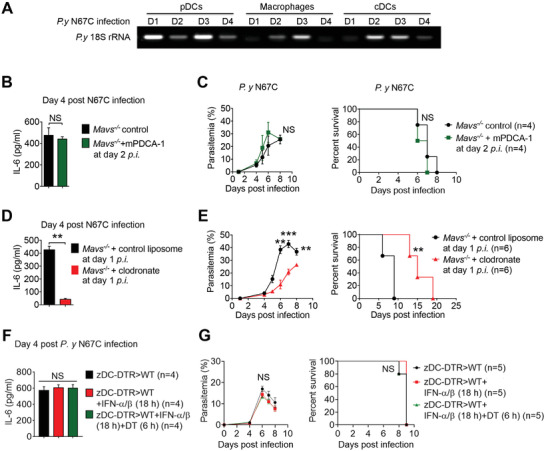
Macrophages are the main source of late IL‐6 and are detrimental for mice survival. A) The cell populations of pDCs, cDCs, and macrophages were isolated from WT mice splenocytes at indicated time points post N67C infection by cell isolation kits, and then analyzed for cell‐specific expression of *P. yoelii* 18S rRNA by PCR. B,C) *Mavs^–/–^* mice (*n* = 4) were infected with N67C, followed by anti‐m‐PDCA‐1 antibody injection at day 2 p.i. Sera were collected at day 4 p.i. and subjected to ELISA analysis of IL‐6 (B). Parasitemia and mortality rates (C) were monitored daily. D,E) *Mavs^–/–^* mice (*n* = 6) were infected with N67C, followed by clodronate liposomes injection at day 1 post infection. Sera were collected at day 4 p.i. and subjected to ELISA analysis of IL‐6 (D). Parasitemia and mortality rates were monitored daily (E). F,G) zDC‐DTR>WT chimeric mice were infected with N67C, followed by administration with or without DT (100 ng) at 6 h p.i. and with 800 U/g IFN‐α/β at 18 h p.i. Sera were collected at day 4 p.i. and subjected to ELISA analysis of IL‐6 (F). Parasitemia and mortality rates were monitored daily (G). Data are representative of three independent experiments and are plotted as the mean ± SD. ^**^ *p*<0.01, ^***^ *p*<0.001 versus corresponding control. NS, not significant.

To determine the primary cellular source of the late IL‐6 appearing after an N67C infection, we isolated pDCs, macrophages, and cDCs from WT mice at different time points after N67C infections. We found that N67C‐specific 18S rRNA was detectable in only the pDCs—not in other cell types—at day one post N67C infection (**Figure** **2**A). However, at days two and three p.i., we detected N67C‐parasite nucleic acids in the pDCs, macrophages, and cDCs (Figure 2A), suggesting that N67C could be detected by pDCs and then macrophages and cDCs. Considering previous findings that pDCs are the major cell population of early type‐I IFN production after YM infections,^[^ ^16^ ^]^ we then performed a pDC‐depletion experiment by using anti‐mPDCA‐1 antibodies administered at 12 h before and post infection. We found that the pDCs depletion in the *Tmem173^gt^* mice, with a markedly decreased production of type‐I IFN at day one post N67C infection (Figure S2A,D, Supporting Information) and markedly increased parasitemia levels and a shortened survival time in the *Tmem173^gt^* mice infected with lethal N67C (Figure S2B,C, Supporting Information). This suggested that the early production of type‐I IFN cytokines by pDCs is important and required for a partial resistance to N67C infections. Moreover, the depletion of the macrophages at 12 h before and after the infections had no effects on the production of type‐I IFN cytokines, as previously demonstrated.^[^ ^16^ ^]^

To identify the cellular source of the late‐stage IL‐6 production, we then depleted either pDCs or macrophages (Figure S2E, Supporting Information) in the *Mavs^–/–^* mice (which produce large amounts of early IFN‐α/β and late IL‐6) after lethal N67C infections. We found that the pDC depletion did not affect the IL‐6 production at day four, nor did it affect the mouse survival time (Figure 2B,C), indicating that pDCs do not contribute to the late‐stage IL‐6 production (at day four p.i.). In contrast, we did find that the macrophage depletion at day one post N67C infection markedly decreased the IL‐6 serum levels of the sensitive strain (*Mavs^–/–^*) mice at day four post N67C infection (Figure 2D). Furthermore, the depletion of macrophages decreased the parasitemia levels in the *Mavs^–/–^* mice and enhanced their resistance to N67C infections (Figure 2E). These results suggest that macrophages are the primary source of IL‐6 production at day four upon N67C infection.

To further investigate whether cDCs are involved in the late IL‐6 production, we generated zinc‐figure transcription‐factor‐driven DTR expression in cDC (zDC‐DTR) bone marrow chimeric mice (zDC‐DTR > WT). We showed that diphtheria toxin (DT for short) injections into the zDC‐DTR bone marrow chimeras resulted in a specific depletion of cDCs in 24 h and maintained the depletion for 4 days (Figure S2F, Supporting Information). Next, we treated the zDC‐DTR chimeric mice with the DT at 6 h p.i. and found that the cDC depletion had no effect on the late IL‐6 production at day four p.i. (Figure 2F). Moreover, when the DT‐treated chimeric mice were pre‐treated with IFN‐α/β, they consistently showed parasitemia levels and survival similar to those of control groups (DT‐untreated chimeric mice with pretreatments of IFN‐α/β) (Figure 2G), suggesting that cDCs are not the main source of the late IL‐6 production.

### 2.3 Late IL‐6 Inhibits Protective Immunity against N67C Infections

**Figure 3:**
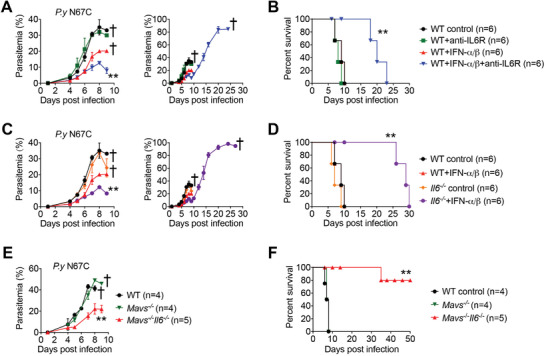
Opposite roles of early IFN‐α/β and late IL‐6 in generating immunity against N67C infection. A,B) WT mice (*n* = 6) were infected with N67C, followed by administration of 800 U/g IFN‐α/β at 18 h and/or blockage of IL‐6R with anti‐IL6R antibody (500 µg) at day 3 p.i. Parasitemia (A) and mortality rates (B) were monitored daily. C,D) WT and *Il6^–/–^* mice (*n* = 6) were infected with N67C, followed by administration of 800 U/g IFN‐α/β or control BSA at 18 h p.i. Parasitemia (C) and mortality rates (D) were monitored daily. E,F) WT, *Mavs^–/–^* and *Mavs^–/–^Il6^–/–^* mice were infected with N67C. Parasitemia (E) and mortality rates (F) were monitored daily. Data are representative of three independent experiments and are plotted as the mean ± SD. ^**^ *p* < 0.01, ^***^ *p* < 0.001 versus corresponding control. † denotes mouse death.

To determine the potential functions of early IFN‐α/β and late IL‐6 in regulating immunity against N67C infections, we performed experiments using N67C‐infected WT mice divided into four different treatment groups. We found that a combination of an early recombinant IFN‐α/β injection at 18 h p.i. with an administration of anti‐IL‐6R antibodies at day three p.i. could reduce parasitemia and prolong mouse survival (**Figure** **3**A,B). In contrast, WT mice treated with a recombinant IFN‐α/β at 18 h or anti‐IL6R antibodies at day three p.i. failed to generate strong immunity, leading to high parasitemia levels and mortalities within 10 days p.i. (Figure 3A,B). Moreover, *Il6^–/–^* mice were sensitive to N67C infections, with respect to parasitemia levels and mortalities, compared to the WT mice (Figure 3C,D). However, N67C‐infected *Il6^–/–^* mice treated with IFN‐α/β at 18 h p.i. had markedly reduced parasitemia levels and increased survival rates compared with N67C‐infected WT mice (Figure 3C,D), suggesting that both an early IFN‐α/β treatment and the blockage of late IL‐6 production are necessary and sufficient to generate strong immunity against N67C infections.

Following this, we aimed to define the detrimental role of late IL‐6 in anti‐malaria immunity. We infected *Mavs^–/–^* mice (which produce both large amounts of early IFN‐α/β and late IL‐6) with N67C and treated them with anti‐IL6R antibodies at day three p.i. We found comparably low parasitemia levels and mortalities compared with control antibody‐treated *Mavs^–/–^* mice (Figure S3A,B, Supporting Information). Although an early exogenous IFN‐α/β treatment and the blockage of late IL‐6 production can enhance anti‐malaria immunity, such immunity confers only a partial resistance to N67C infections; mice died at 25–30 days post N67C infection. We reasoned that this partial resistance to N67C infections may be due to an insufficient early IFN‐α/β treatment or an incomplete blockage of IL‐6 signaling. To test this possibility, we generated *Mavs^–/–^Il6^–/–^* DKO mice by crossing *Mavs^–/–^* mice with *Il6^–/‐^* mice and found that the DKO mice generated strong immunity against N67C infections with lower parasitemia levels and markedly prolonged mouse survival compared to WT and *Mavs^–/–^* mice (Figure 3E,F). Notably, the *Mavs^–/–^Il6^–/–^* DKO mice were almost completely resistant to the lethal N67C infections. Taken together, these results suggest that both an early robust IFN‐α/β production and the blockage of late IL‐6 production are essential for generating adequate protective immunity against N67C infections.

### 2.4 cGAS and STING Interact with MyD88 and Induce IL‐6 Production through MyD88‐p38 Signaling

**Figure 4:**
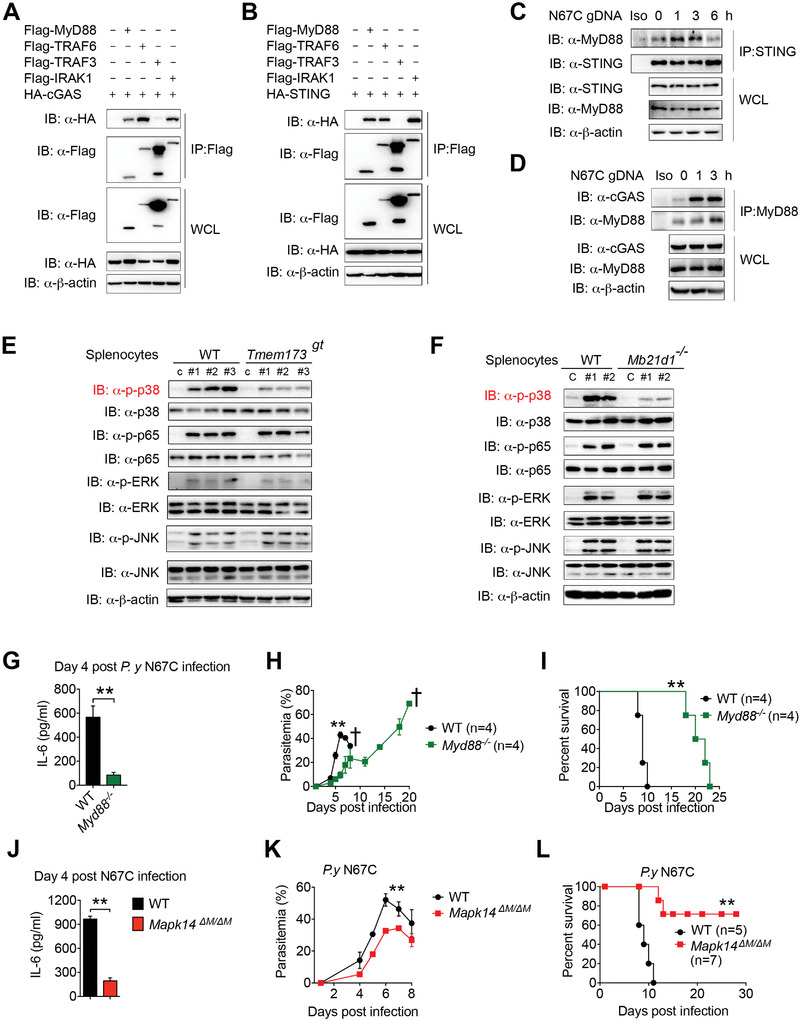
cGAS/STING interact with MyD88 and trigger MAPK/p38 dependent IL‐6 production. A) 293T cells were transfected with HA‐cGAS together with Flag‐MyD88, Flag‐TRAF6, Flag‐TRAF3, or Flag‐IRAK1. Flag‐tagged proteins were immunoprecipitated with anti‐Flag beads followed by anti‐HA immunoblotting. B) 293T cells were transfected with HA‐STING together with Flag‐MyD88, Flag‐TRAF6, Flag‐TRAF3, or Flag‐IRAK1. Flag‐tagged proteins were immunoprecipitated with anti‐Flag beads followed by anti‐HA immunoblotting. C) RAW264.7 cells were treated with N67C gDNA and the cell lysates were collected at the indicated time points, then used for immunoprecipitation with anti‐STING, followed by immunoblotting with the indicated antibodies. Iso stands for isotype IgG control. D) RAW264.7 cells were treated with N67C gDNA and the cell lysates were collected at the indicated time points, then used for immunoprecipitation with anti‐MyD88, followed by immunoblotting with the indicated antibodies. E) WT and *Tmem173^gt^* mice (*n* = 3) were infected with N67C and splenocytes were collected at day 3 p.i., and uninfected mice served as control. Cell lysates were analyzed by immunoblotting with the indicated antibodies. F) WT and *Mb21d1^–/–^* mice (*n* = 2) were infected with N67C and splenocytes were collected at day 3 p.i., and uninfected mice served as control. Cell lysates were analyzed by immunoblotting with the indicated antibodies. G–I) WT and *Myd88^–/–^* mice (*n* = 4) were intraperitoneally infected with N67C. Sera were collected at the indicated time points p.i. and subjected to ELISA analysis of IL‐6 (G), and parasitemia (H) and mortality rates (I) were monitored daily. J–L) WT (*n* = 5) and *Mapk14^ΔM/ΔM^* (*n* = 7) mice were intraperitoneally infected with N67C. Sera were collected at indicated time points p.i. and subjected to ELISA analysis of IL‐6 (J), and parasitemia (K) and mortality rates (L) were monitored daily. IP, IB, and WCL denote immunoprecipitation, immunoblotting, and whole cell lysate, respectively. Data are representative of three independent experiments and are plotted as the mean ± SD. ^**^ *p* < 0.01 versus corresponding control. † denotes mouse death.

Next, we sought to elucidate the mechanisms by which cGAS/STING triggers IL‐6 production in macrophages. We hypothesized that cGAS/STING might cross‐talk with MyD88 or other signaling molecules in the NF‐κB and MAPK signaling for IL‐6 production. To test this possibility, we performed a co‐immunoprecipitation of 293T cells expressing cGAS or STING plus MyD88, TRAF3, TRAF6, or interleukin‐1 receptor‐associated kinase 1 (IRAK1), and we found that both cGAS and STING could interact with MyD88, in addition to TRAF6 and IRAK1 (**Figure** **4**A,B). Endogenous experiments also showed that MyD88 interacts with cGAS and STING upon an N67C gDNA stimulation in a time‐dependent manner (Figure 4C,D). Since MyD88 can induce the production of proinflammatory cytokines through either the NF‐κB pathway or the MAPK pathway,^[^ ^15^ ^]^ we then determined which pathway is required for a cGAS/STING/MyD88‐induced late IL‐6 production. We infected sensitive (WT) and resistant (*Tmem173^gt^* and *Mb21d1^–/–^*) strains of mice with N67C, collected splenocytes at day three p.i., and compared the signaling cascade activations. We found that the phosphorylation of p65, extracellular signal‐regulated kinase (ERK), and c‐Jun N‐terminal kinase (JNK) was similar in the WT, *Tmem173^gt^* and *Mb21d1^–/–^* splenocytes (Figure 4E,F). In contrast, p38 phosphorylation was much weaker in the *Tmem173^gt^* and *Mb21d1^–/–^* splenocytes compared with the WT splenocytes (Figure 4E,F). Furthermore, there was no appreciable difference in p38 phosphorylation between N67C RNA‐stimulated WT and *Mavs^–/‐^* bone marrow‐derived macrophages (BMDMs) (Figure S4A, Supporting Information). These results suggest that p38/MAPK signaling activation is dependent on cGAS/STING but not on the MAVS signaling pathway during N67C infections. To determine whether TLR‐MyD88 signaling can induce the late IL‐6 production, we showed that the production in *Tlr7^–/–^* and *Tlr9^–/–^* mice was similar to that in WT mice (Figure S4B, Supporting Information), suggesting that TLR7 and TLR9 are not the major signaling pathways involved in late IL‐6 production and anti‐malaria immunity in the N67C infection model. These data are consistent with the fact that TLR7 and TLR9 are mainly expressed in pDCs^[^ ^32^ ^]^ but not in macrophages, and the depletion of pDCs has no effect on the late IL‐6 production. Furthermore, like the *Tmem173^gt^* and *Mb21d1^–/‐^* mice, the *Myd88^–/–^* mice presented a markedly reduced late IL‐6 release and reduced parasitemia levels, thus prolonging their survival (Figures 1C,D and 4G–I; Figure S4B, Supporting Information). These results suggest that the cooperation between cGAS/STING and the MyD88 (but not TLR) signaling pathways is required for the late IL‐6 production.

We also found that p38 phosphorylation increased in the spleen, liver, and peripheral blood (Figure S4C, Supporting Information). Indeed, the expression levels of IL‐6 were upregulated in the tissues after N67C infections (Figure S4D, Supporting Information). To directly test the role of p38 signaling in IL‐6 production, we then generated *Mapk14^ΔM/ΔM^* mice by crossing *Mapk14^f/f^* with *LysM‐Cre* mice and challenged them with lethal N67C infections. We found that the *Mapk14^ΔM/ΔM^* mice produced very low levels of IL‐6 at day four p.i. and markedly reduced parasitemia levels (Figure 4J,K). Most importantly, more than 70% of the *Mapk14^ΔM/ΔM^* mice were resistant to N67C infections and had a prolonged survival compared with WT mice (Figure 4L). Taken together, these results clearly indicate that the cGAS‐STING‐MyD88‐p38 signaling pathway is responsible for the late IL‐6 production, which dampens anti‐N67C immune responses.

### 2.5 Late IL‐6 Signaling Inhibits the Host Immunity against N67C Infections by Suppressing the T Cell Function

**Figure 5:**
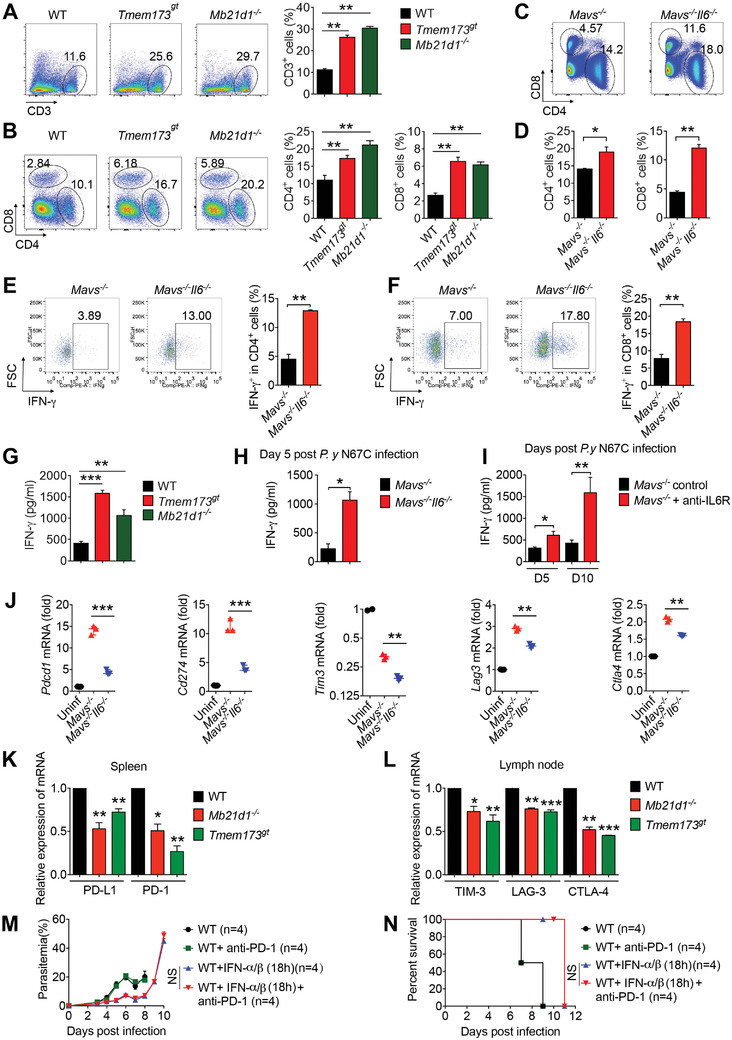
Late IL‐6 signaling inhibits host immunity against N67C infection by suppressing T cell function. A,B) WT, *Tmem173^gt^* and *Mb21d1* ^–/–^ mice (*n* = 3) were infected with N67C. Splenocytes were collected at day 5 p.i., and subjected to FACS analysis of CD3^+^ cells (A), CD4^+^ cells and CD8^+^ cells in splenocytes (B). C,D) *Mavs^–/‐^* and *Mavs^–/–^Il6^–/–^* mice (*n* = 3) were infected with N67C. Splenocytes were collected at day 5 p.i., and subjected to FACS analysis of CD4^+^ cells and CD8^+^ cells in splenocytes. E,F) *Mavs^–/‐^* and *Mavs^–/–^Il6^–/–^* mice (*n* = 3) were infected with N67C. Splenocytes were collected at day 5 p.i. and stimulated with N67C crude antigen in vitro, then subjected to FACS analysis of IFN‐γ^+^ cells in CD4^+^ cells (E) and in CD8^+^ cells (F). G) WT, *Tmem173^gt^*, and *Mb21d1^–/–^* mice (*n* = 3) were infected with N67C. Sera were collected at day 5 p.i. and subjected to ELISA analysis of IFN‐γ. H) *Mavs^–/‐^* and *Mavs^–/–^Il6^–/–^* mice (*n* = 3) were infected with N67C. Sera were collected at day 5 p.i. and subjected to ELISA analysis of IFN‐γ. I) *Mavs^–/–^* mice (*n* = 3) were infected with N67C, and then treated with or without anti‐IL6R antibody at day 2 p.i. Sera were collected at indicated time points and subjected to ELISA analysis of IFN‐γ. J) *Mavs^–/‐^* and *Mavs^–/–^Il6^–/–^* mice (*n* = 3) were infected with N67C. Splenocytes were collected at day 5 p.i. RNAs from splenocytes were isolated and used for expression analysis using qPCR. K, L) WT, *Tmem173^gt^*, and *Mb21d1^–/–^* mice (*n* = 3) were infected with N67C. Splenocytes (K) and lymph nodes (L) were collected at day 5 p.i. RNAs from splenocytes and lymph nodes were isolated and used for expression analysis using qPCR. M,N) WT mice (*n* = 4) were infected with N67C, followed with administration of recombinant IFN‐α/β or control BSA at 18 h p.i., then treated with anti‐PD‐1 antibody. Parasitemia (M) and mortality rates (N) were monitored daily. Data are representative of three independent experiments and are plotted as the mean ± SD. ^*^ *p* < 0.05, ^**^ *p* < 0.01, ^***^ *p* < 0.001 versus corresponding control.

To understand how late IL‐6 dampens anti‐malaria immunity, we considered whether IL‐6 negatively regulates T cell immunity. For this reason, we compared T cell population changes at day five for WT (sensitive), *Tmem173^gt^* (resistant), and *Mb21d1^–/‐^* (resistant) mice. We found that the percentages of CD3^+^, CD4^+^, and CD8^+^ cells increased in the splenocytes of the *Tmem173^gt^* and *Mb21d1^–/‐^* mice compared with the WT mice after the N67C infections (**Figure** **5**A,B). Similar results were obtained when comparing *Mavs^–/–^Il6^–/–^* (resistant) mice with *Mavs^–/–^* (sensitive) mice infected with N67C (Figure 5C,D). Next, we considered whether T cell function is affected by the late IL‐6 production. We found that IFN‐γ^+^ CD4^+^ T cells and IFN‐γ^+^ CD8^+^ T cell populations increased in the splenocytes of *Mavs^–/–^Il6^–/–^* mice and anti‐IL6R antibody‐treated *Mavs^–/–^* mice compared with those in *Mavs^–/–^* mice infected with N67C (Figure 5E,F; Figure S5A, Supporting Information). Furthermore, we found that the mRNA levels of *Ifng* in splenocytes and T cells, as well as the serum cytokine levels of IFN‐γ, were markedly increased in *Tmem173^gt^*, *Mb21d1^–/–^*, *Mavs^–/–^Il6^–/–^*, and anti‐IL6R antibody‐treated *Mavs^–/–^* mice infected with N67C, compared with the levels in WT and *Mavs^–/–^* mice infected with N67C (Figure 5G–I; Figure S5B–D, Supporting Information). This suggests that the late IL‐6 signaling inhibits T cell function for IFN‐γ release at the late stage after an N67C infection.

To understand how late IL‐6 signaling inhibits T cell immunity, we then examined T cell suppression and exhaustion surface markers and found that PD‐1^+^ cells were decreased in the splenocytes and peripheral blood of *Mavs^–/–^Il6^–/–^* or anti‐IL‐6R antibody‐treated *Mavs^–/–^* mice, compared with those in N67C‐infected *Mavs^–/–^* mice (Figure S5E–G, Supporting Information). Moreover, the expression of PD‐1, CTLA‐4 in CD8^+^ T cells and LAG‐3 in CD4^+^ and CD8^+^ T cells was markedly reduced in *Mavs^–/–^Il6^–/–^* mice compared with the T cells of *Mavs^–/–^* mice infected with N67C (Figure S5H, Supporting Information). The mRNA levels of *Pdcd1* were also consistently markedly increased in T cells from the spleens of *Mavs^–/–^* mice compared with T cells from the spleens of *Mavs^–/–^Il6^–/–^* mice (Figure S5I, Supporting Information). These results suggest that the late IL‐6 signaling after the N67C infections increases the expression of suppressive markers on T cells. Similarly, we found that the mRNA levels of *Pdcd1* (PD‐1), *Cd274* (PD‐L1)*, Tim3, Lag3*, and *Ctla4* were significantly increased in the splenocytes and lymph nodes of WT and *Mavs^–/–^* mice compared with those in *Tmem173^gt^*, *Mb21d1^–/–^*, and anti‐IL‐6R‐treated *Mavs^–/–^* mice after N67C infections (Figure 5J–L; Figure S5J, Supporting Information). Taken together, these results suggest that the deletion or blockage of IL‐6 production enhances the protective immunity against N67C infections by inhibiting the expression of suppressive or negative signaling molecules.

Following this, we aimed to directly test whether a blockade PD‐1 signaling with anti‐PD‐1 antibodies can enhance anti‐malaria immunity. We treated N67C‐infected WT mice with or without early IFN‐α/β at 18 h p.i., followed by either an anti‐PD‐1 or a control antibody treatment. We found that there were no differences in the parasitemia levels and survival rates of the N67C‐infected WT mice treated with control antibodies and the anti‐PD‐1 antibody mice (Figure 5M,N). Similarly, we did not observe any differences in the parasitemia levels and mouse survival of N67C‐infected IFN‐α/β‐treated WT mice with or without anti‐PD‐1 treatments (Figure 5M,N). Early IFN‐α/β treatments reduced parasitemia levels and slightly (but not significantly) prolonged the mouse survival (Figure 5M,N). These results suggest that a combination of early IFN‐α/β and anti‐PD‐1 antibody treatments may not reduce parasitemia levels and improve mouse survival rates, even though late IL‐6 inhibits T cell immunity.

### 2.6 IL‐6 Induces a CD11b^+^Ly6C^hi^ Proinflammatory Monocyte Expansion and Inhibits T Cell Function

**Figure 6:**
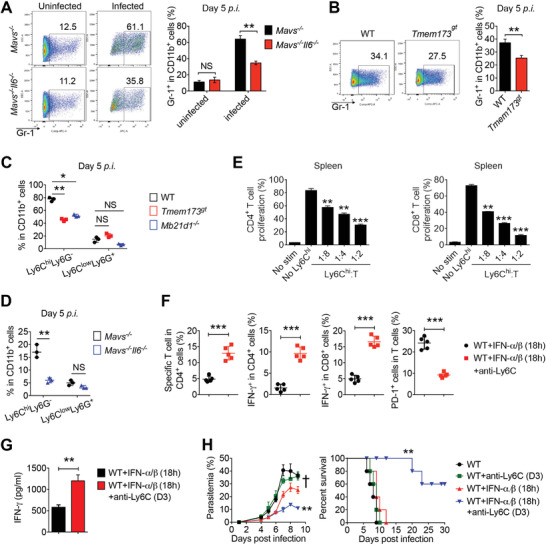
IL‐6 induces CD11b^+^Ly6C^hi^ proinflammatory monocytes expansion and inhibits T cell function. A) *Mavs^–/‐^* and *Mavs^–/–^Il6^–/–^* mice (*n* = 3) were infected with N67C. Splenocytes were collected at day 5 p.i., and subjected to FACS analysis of Gr‐1^+^ cells in CD11b^+^ cells. B) WT and *Tmem173^gt^* mice (*n* = 3) were infected with N67C. Splenocytes were collected at day 5 p.i., and subjected to FACS analysis of Gr‐1^+^ cells in CD11b^+^ cells. C) WT, *Tmem173^gt^*, and *Mb21d1^–/–^* mice (*n* = 3) were infected with N67C. Splenocytes were collected at day 5 p.i., and subjected to FACS analysis of Ly6C^hi^Ly6G^–^ cells and Ly6C^lo^Ly6G^+^ cells in CD11b^+^ cells. D) *Mavs^–/‐^* and *Mavs^–/–^Il6^–/–^* mice (*n* = 3) were infected with N67C. Splenocytes were collected at day 5 p.i., and subjected to FACS analysis of Ly6C^hi^Ly6G^–^ cells and Ly6C^lo^Ly6G^+^ cells in CD11b^+^ cells. E) Proliferation of CD4^+^ and CD8^+^ cells stimulated with CD3/CD28 antibody in the presence of Ly6C^hi^ cells isolated from spleens of N67C infected WT mice (*n* = 3). F,G) WT mice (*n* = 5) were infected with N67C, followed by administrated with recombinant IFN‐α/β at 18 h p.i., and then treated with or without anti‐Ly6C antibody (rat IgG serves as control) at day 3 p.i. Splenocytes were collected at day 6 p.i., and subjected to FACS analysis of malaria specific T cells %, PD‐1^+^ cells %, IFN‐γ^+^ cells in indicated cell populations (F). Sera were collected at day 6 p.i. and subjected to ELISA analysis of IFN‐γ (G). H) WT mice (*n* = 5) were infected with N67C, followed by administrated with or without recombinant IFN‐α/β at 18 h p.i., and then treated with or without anti‐Ly6C antibody at day 3 p.i., parasitemia and mortality rates were monitored daily. Data are representative of three independent experiments and are plotted as the mean ± SD. ^*^ *p* < 0.05, ^**^ *p* < 0.01, ^***^ *p* < 0.001 versus corresponding control. NS, not significant. † denotes mouse death.

Since a blockade of PD‐1/PD‐L1 signaling could not restore anti‐malaria immunity, we reasoned that late IL‐6 may regulate other immune‐cell populations. Therefore, we expanded our research to suppressive immune cells, such as T‐regulatory cells (Tregs), DCs, macrophages, and monocytes. We found no differences in the Treg cells (Figure S6A, Supporting Information) and DCs (CD86^+^ CD11c^+^) (Data not shown) of *Mavs^–/–^* and *Mavs^–/–^Il6^–/–^* mice, suggesting that Treg cells and DCs are not responsible for inhibiting the protective immunity against N67C infections. Since proinflammatory monocytes and myeloid‐derived suppressive cells (MDSCs) have been reported to inhibit immunity in cancer and infectious diseases,^[^ ^33^, ^34^, ^35^, ^36^ ^]^ we then examined the Gr‐1^+^ (Ly6C/Ly6G) cells in N67C‐sensitive and N67C‐resistant mice. We found that the percentages of Gr‐1^+^ cells in the CD11b^+^‐gated cell populations in the splenocytes of *Mavs^–/–^* (sensitive) mice were much higher than those of *Mavs^–/–^Il6^–/–^* (resistant) mice after N67C infections, while the basal levels of the Gr‐1^+^ (Ly6C/Ly6G) cells were similar between *Mavs^–/–^* and *Mavs^–/–^Il6^–/–^* mice (**Figure** **6**A). This suggests that late IL‐6 at day four p.i. may induce Gr‐1^+^ (Ly6C/Ly6G) cell expansions. Similar results were obtained using splenocytes from WT and *Tmem173^gt^* (partially resistant) mice (Figure 6B). The phosphorylation of signal transducer and activator of transcription 3 (Stat‐3) was much weaker in the *Tmem173^gt^* (partially resistant) mice compared to the WT mice (Figure S6B, Supporting Information).

To determine which subset of Gr‐1^+^ (Ly6C/Ly6G) is expanded by IL‐6 production during N67C infections, we analyzed the CD11b^+^Ly6C^lo^Ly6G^+^ and CD11b^+^Ly6C^hi^Ly6G^–^ cells in WT mice after N67C infections (Figure S6C, Supporting Information). We found that the percentages of CD11b^+^Ly6C^hi^Ly6G^–^ cells were markedly increased from day four to day six p.i. (Figure S6D, Supporting Information), while the percentages of CD11b^+^Ly6C^lo^Ly6G^+^ cells did not change significantly during the N67C infections (Figure S6E, Supporting Information). Moreover, the percentages of CD11b^+^Ly6C^hi^Ly6G^–^ cells were much lower in *Tmem173^gt^*, *Mb21d1^–/–^*, *Myd88^–/–^*, and *Mavs^–/–^Il6^–/–^* mice than in WT and *Mavs^–/–^* mice at day five after N67C infection (Figure 6C,D; Figure S6F, Supporting Information). In contrast, we did not observe any appreciable difference in the percentages of the CD11b^+^Ly6C^lo^Ly6G^+^ cells of resistant (*Tmem173^gt^*, *Mb21d1^–/–^*, *Myd88^–/–^*, and *Mavs^–/–^Il6^–/–^*) and sensitive (WT and *Mavs^–/–^*) mice (Figure 6C,D; Figure S6F, Supporting Information), suggesting that late IL‐6 mainly induces and expands CD11b^+^Ly6C^hi^Ly6G^–^ proinflammatory monocytes to dampen anti‐malaria immune responses. Thus, to determine whether IL‐6‐induced CD11b^+^Ly6C^hi^Ly6G^–^ proinflammatory monocytes inhibit T cell function and protective immunity, we isolated CD11b^+^Ly6C^hi^Ly6G^–^ cells at day five post N67C infection in WT mice, and then co‐cultured them with CFSE‐labeled naïve CD3^+^ T cells from splenocytes (Figure S6G, Supporting Information). We found that the proliferative activities of CD4^+^ and CD8^+^ T cells stimulated with CD3/CD28 antibodies were inhibited in the presence of CD11b^+^Ly6C^hi^ cells from the spleens of N67C‐infected WT mice (Figure 6E).

Next, to determine the functional relevance of CD11b^+^Ly6C^hi^ proinflammatory monocytes, we administered recombinant IFN‐α/β to WT mice at 18 h p.i., and then depleted CD11b^+^Ly6C^hi^ proinflammatory monocytes by injecting anti‐mouse Ly6C antibodies at day three post N67C infection (Figure S6H, Supporting Information). We found that the percentages of malaria‐specific and IFN‐γ‐producing T cells and IFN‐γ serum amounts increased while PD‐1^+^ T cells decreased after the monocyte depletion (Figure 6F,G). Moreover, a depletion of CD11b^+^Ly6C^hi^ cells by an anti‐Ly6C treatment at day three post N67C infection markedly reduced the parasitemia levels and prolonged the survival of WT mice that received early IFN‐α/β treatments at 18 h p.i., compared with WT mice that received either early IFN‐α/β treatments or an anti‐Ly6C treatment alone (Figure 6H). Taken together, these results suggest that late IL‐6 induces and expands CD11b^+^Ly6C^hi^ proinflammatory monocytes, which in turn mediate the inhibition of adaptive immune responses against lethal malaria N67C infections; the depletion of CD11b^+^Ly6C^hi^ proinflammatory monocytes restores the anti‐malaria immunity and prolongs mouse survival.

### 2.7 The Negative Regulatory Mechanism of Late IL‐6 is Generally Applicable to Infections of Other Lethal Malaria Strains

**Figure 7:**
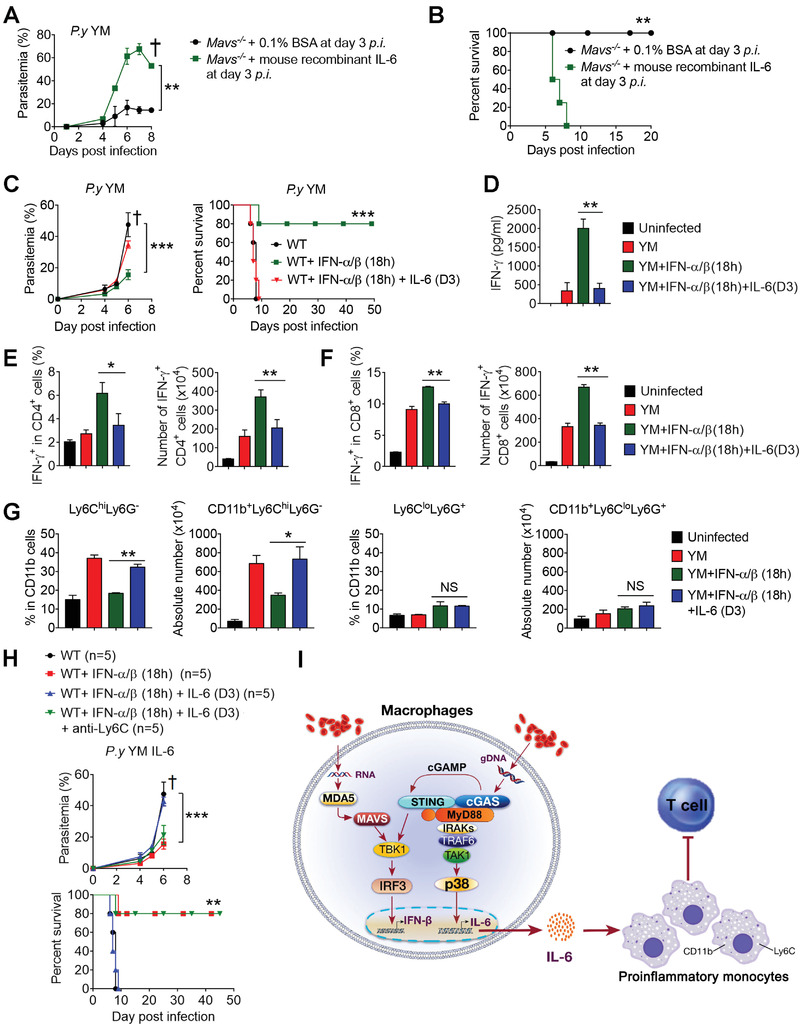
Administration of IL‐6 is detrimental for host generating immune responses against YM infection. A,B) *Mavs^–/–^* mice (*n* = 4) were infected with YM, and then treated with or without recombinant IL‐6 at day 3 p.i., parasitemia (A) and survival (B) were monitored daily. C) WT mice (*n* = 5) were infected with YM, followed by administrated with recombinant IFN‐α/β at 18 h p.i., and then treated with recombinant IL‐6 at day 3 p.i. The parasitemia and survival were monitored daily. D–F) WT mice (*n* = 5) were infected with YM, followed by administrated with recombinant IFN‐α/β at 18 h p.i., and then treated with recombinant IL‐6 at day 3 p.i. Sera were collected at day 5 p.i. and subjected to ELISA analysis of IFN‐γ (D). Splenocytes were collected at day 5 p.i., and subjected to FACS analysis of IFN‐γ^+^ cells in CD4^+^ cells (E) and in CD8^+^ cells (F). G) WT mice (*n* = 5) were infected with YM, followed by administrated with recombinant IFN‐α/β at 18 h p.i., and then treated with recombinant IL‐6 at day 3 p.i. Splenocytes were collected at day 5 p.i., and subjected to FACS analysis of Ly6C^hi^Ly6G^–^ cells and Ly6C^lo^Ly6G^+^ cells in CD11b^+^ cells. H) WT mice (*n* = 5) were infected with YM, followed by administration with or without recombinant IFN‐α/β at 18 h p.i., and then treated with recombinant IL‐6 at day 3 p.i., followed by treatment with or without anti‐Ly6C antibody every two days starting from day 3 p.i. Parasitemia and mortality rates were monitored daily. I) A schematic model to show that activation of cGAS‐STING by *Plasmodium* N67C recruits MyD88 to induce p38 MAPK mediated IL‐6 production in macrophages at late stage of infection, which further induces and expands CD11b^+^Ly6C^hi^ proinflammatory monocytes to inhibit T cell proliferation, function and host anti‐malaria immune responses against N67C infection. Data are representative of three independent experiments and are plotted as the mean ± SD. ^*^ *p* < 0.05, ^**^ *p* < 0.01, ^***^ *p* < 0.001 versus corresponding control. † denotes mouse death.

Following this, we considered whether delayed IL‐6‐induced CD11b^+^Ly6C^hi^ cell‐mediated immunosuppressive mechanisms can be generally applied to other lethal malaria infections. To this end, we used YM‐resistant *Mavs^–/–^* mice as a model. We treated YM infected‐*Mavs^–/–^* mice with IL‐6 at day three p.i. and found that the treatment markedly increased the mice parasitemia levels and resulted in a 100% death percentage, while BSA control‐treated mice survived (**Figure** **7**A,B), suggesting that a late IL‐6 treatment markedly inhibits the anti‐malaria immunity against YM infections. We also found that the YM‐infected *Mavs^–/–^* mice with late IL‐6 treatment showed markedly reduced CD3^+^, CD4^+^, and CD8^+^ T cell populations in the peripheral blood and spleens, compared with the control mice (Figure S7A–C, Supporting Information). Moreover, analysis of intracellular cytokine staining of T cells revealed that IFN‐γ‐producing CD4^+^ and CD8^+^ T cells were markedly reduced in the YM‐infected *Mavs^–/–^* mice treated with IL‐6 compared with control mice (Figure S7D,E, Supporting Information). Consistent with these observations, we found that serum levels of IFN‐γ and mRNA levels of *Ifng* in splenocytes were markedly decreased after IL‐6 treatment in YM‐infected *Mavs^–/–^* mice (Figure S7F, Supporting Information). Conversely, *Cd274* (PD‐L1) expressions in the splenocytes of YM‐infected *Mavs^–/–^* mice were markedly increased after the IL‐6 treatment was administered (Figure S7G, Supporting Information).

To further investigate this finding in WT mice, we generated resistant WT mice through the early administration of IFN‐α/β (at 18 h p.i.), which either was or was not followed by a late‐IL‐6 treatment at day three post YM infection. It was found that the parasitemia levels and mortality rates were markedly increased when the mice were treated with late IL‐6 (Figure 7C). Consistent with this observation, IFN‐γ serum levels and either IFN‐γ‐producing CD4^+^ or IFN‐γ‐producing CD8^+^ T cells were markedly reduced in YM‐infected, IFN‐α/β‐administered WT mice after they were treated with late IL‐6, compared with WT control mice that received only early IFN‐α/β treatments alone (Figure 7D–F). Mechanistically, the late IL‐6 treatment induced and expanded the CD11b^+^Ly6C^hi^Ly6G^–^ proinflammatory monocytes but not the CD11b^+^Ly6C^lo^Ly6G^+^ cells in the YM infection model (Figure 7G). Moreover, the depletion of the CD11b^+^Ly6C^hi^ cells by the anti‐Ly6C antibody treatment at day three post YM infection markedly reduced the parasitemia levels and prolonged the survival of the WT mice that received early IFN‐α/β and late IL‐6 treatments (Figure 7H). Taken together, these results indicate that through the induction and expansion of CD11b^+^Ly6C^hi^ proinflammatory monocytes, the late IL‐6‐mediated immune suppression is a general mechanism that can be used to intervene in infections of different lethal strains of malaria (Figure 7I).

## 3 Discussion and Conclusion

cGAS and MDA5 are responsible for sensing *Plasmodium* gDNA and RNA, triggering STING/MAVS‐mediated type‐I IFN signaling and the upregulation of IRF3 target genes, including ISGs and negative genes.^[^ ^16^, ^17^, ^19^, ^26^, ^37^, ^38^, ^39^ ^]^ The early production of type‐I IFN increases in cGAS‐, STING‐, MDA5‐, and MAVS‐deficient mice, regardless whether they have an N67C or YM infection, mainly through a mechanism by which STING and MAVS signaling induces negative regulators (such as SOCS1) to inhibit the Myd88‐dependent type‐I IFN production in pDCs.^[^ ^16^ ^]^ Furthermore, an early robust production of IFN‐α/β protects hosts from malaria parasites and virus infections.^[^ ^9^, ^16^, ^22^, ^40^, ^41^ ^]^ However, MDA5‐ and MAVS‐deficient mice fail to resist N67C infections, even with the early, robust production of IFN‐α/β, indicating that other suppressive mechanisms operate during N67C infections but not during YM infections.

Due to differences between the pathogenicity of plasmodium parasites and host immune responses, infections of different lethal malaria strains cause diverse symptoms, including different cytokine‐production patterns, immune‐cell responses, and host resistances, which dictate strain‐specific immune responses and lethalities.^[^ ^8^, ^9^, ^42^ ^]^ *P. y. nigeriensis* N67 induces early type‐I IFN signaling to suppress parasitemia levels, while its isogenic strain *P. y. nigeriensis* N67C triggers the p38 signaling pathway, leading to host death.^[^ ^9^ ^]^ However, the underlying mechanisms remain largely unknown. In this paper, we showed that upon the occurrence of an N67C infection, a late IL‐6 production in the macrophages of WT mice is the key cytokine factor that induces and expands CD11b^+^Ly6C^hi^ proinflammatory monocytes to inhibit the proliferation and function of T cells as well as the host anti‐malaria immune response against the N67C infection. Beside its role in type‐I IFN signaling, cGAS‐STING signaling also activates canonical and non‐canonical NF‐κB, MAPK signaling and STAT transcription factors in immune and tumor cells.^[^ ^14^, ^43^, ^44^, ^45^, ^46^, ^47^ ^]^ Recently, it was reported that cGAS‐STING synergizes with the MyD88 pathway in Ly6C^hi^ monocytes to mediate a late‐stage IFN‐γ production in the lungs upon the occurrence of a *Streptococcus pneumoniae* infection.^[^ ^48^ ^]^ In this study, we showed that cGAS and STING interact with MyD88 and drive the p38‐mediated IL‐6 production in macrophages to impair anti‐N67C immunity. To the best of our knowledge, this is the first study that shows cGAS/STING signaling orchestrates the production of IL‐6 in a MyD88‐p38‐dependent manner during malaria infections. By contrast, MDA5/MAVS signaling pathway does not stimulate p38/MAPK activation by N67C. Using genetic ablations (IL‐6 KO and myeloid‐specific p38 KO mice) and proinflammatory macrophage depletions with antibody (anti‐Ly6C) administrations, this study also shows that the blockage of late IL‐6 production renders mice resistant to lethal N67C infections.

Emerging evidence has suggested that a dysregulated cytokine release is associated with the severity of infectious diseases. For example, the delayed and sustained upregulation of interferon responses have been observed in severe cases of SARS and COVID‐19.^[^ ^49^, ^50^, ^51^ ^]^ A study on a murine model of SARS showed that a robust virus replication is followed by a delayed type‐I IFNs production, which contributes to a dramatic increase in inflammatory monocyte‐macrophages that are responsible for the proinflammatory cytokine release.^[^ ^52^ ^]^ More importantly, an early (not late) administration of type‐I IFNs prior to the peak of virus replication prolongs mouse survival.^[^ ^52^ ^]^ A study on a murine model of MERS showed that an early treatment with rIFN‐β within one day p.i. can protect the host by impairing inflammation and restraining MERS‐CoV‐MA (a mouse‐adapted strain of MERS‐CoV) replications. In contrast, a late IFN‐β treatment fails to provide mice protection and will even result in significantly higher mouse mortality.^[^ ^53^ ^]^ Taken together, these studies suggest that the timing and magnitude of cytokine production dictate the outcomes of infectious diseases.

To understand how the late IL‐6 production mediates immune suppression and impairs anti‐N67C immunity, we first examined whether PD‐1/PD‐L1, CTLA, and TIM3 have roles in inhibiting anti‐malaria immunity. Immune checkpoints, such as PD‐1, CTLA‐4, and TIM‐3, increase in malaria‐infected individuals.^[^ ^54^, ^55^, ^56^, ^57^ ^]^ Moreover, the blockage of either PD‐1 or CTLA‐4 may enhance a T cell activation against malaria infections.^[^ ^58^ ^]^ Although we found that the expression levels of *Pdcd1*, *Cd274*, *Tim3*, *Lag3*, and *Ctla4* were significantly higher in N67C‐sensitive mice (WT and *Mavs^–/–^* mice) compared with N67C‐resistant mice (*Tmem173^gt^*, *Mb21d1^–/–^* and *Mavs^–/–^Il6^–/‐^* mice), the administration of anti‐PD‐1 antibodies failed to provide a resistance to N67C infections. These results suggest that the late IL‐6 production inhibits the anti‐N67C immunity through a different and previously unrecognized mechanism.

IL‐6 has been implicated in the expansion of proinflammatory monocytes, which are known for their capacity to inhibit T cell function and proliferations in cancer and parasite infections.^[^ ^59^, ^60^, ^61^, ^62^, ^63^, ^64^, ^65^, ^66^, ^67^ ^]^ Soluble‐egg Ag (SEA) and schistosome‐worm Ag (SWA) of *S. japonicum* have been reported to promote the accumulation of MDSCs through Janus kinase (JAK)/STAT3 signaling. MDSCs from *S. japonicum*‐infected mice inhibit the proliferation of CD4^+^ and CD8^+^ T cells in vitro in a ROS‐dependent manner. In addition, the study showed that SEA and SWA induce CD11b^+^Ly6G^+^Ly6C^–/low^ MDSCs but not CD11b^+^Ly6G^–^Ly6C^high^ MDSCs.^[^ ^66^ ^]^ CD11b^high^Ly6C^+^ monocytes have been reported to be generated during *P. chabaudi* infections in mice. Furthermore, such monocytes can produce not only inducible nitric‐oxide synthase and reactive‐oxygen intermediates but also phagocytose *P. chabaudi*‐infected erythrocytes.^[^ ^67^ ^]^ However, the negative role and delicate mechanisms of IL‐6 induced proinflammatory monocytes upon the occurrence of a malaria infection are poorly understood. In this study, we provided clear evidence showing the importance of proinflammatory monocytes. We identified a lower percentage of Gr‐1^+^ (Ly6C/Ly6G) cells and CD11b^+^Ly6C^hi^ proinflammatory monocytes in resistant (*Tmem173^gt^*, *Mb21d1^–/–^* and *Mavs^–/–^Il6^–/–^*) mice than in sensitive (WT and *Mavs^–/–^*) mice after N67C infection. Moreover, we showed that CD11b^+^Ly6C^hi^ proinflammatory monocytes isolated from N67C‐infected mice inhibit T cell proliferation in vitro. The depletion of CD11b^+^Ly6C^hi^ proinflammatory monocytes by a specific antibody markedly enhances the T cell function and prolongs survival of N67C‐infected mice. Thus, our results provide direct evidence that CD11b^+^Ly6C^hi^ proinflammatory monocytes are induced by IL‐6 at the late stage of infection and inhibit T cell proliferation and function, thus dampening host anti‐malaria immune response against N67C infections.

To determine whether the late IL‐6‐mediated expansion of CD11b^+^Ly6C^hi^ proinflammatory monocytes operate in other lethal malaria infections, we tested lethal YM malaria infections. The results showed that the administration of late IL‐6 in YM‐infected resistant (e.g., *Mavs^–/–^*) mice at day three p.i. converts the phenotype of resistance to sensitivity. We also showed an induction and expansion of CD11b^+^Ly6C^hi^ proinflammatory monocytes after the administration of late IL‐6. Most importantly, we demonstrated that the depletion of CD11b^+^Ly6C^hi^ proinflammatory monocytes restores the anti‐malaria immunity and prolongs the survival of mice, suggesting a generally applicable mechanism by which late IL‐6‐induced CD11b^+^Ly6C^hi^ proinflammatory monocytes inhibit host immune response to infections of different lethal strains of malaria.

Although there is evidence of the suppression of host anti‐malaria immune responses, the mechanism by which it occurs varies across *Plasmodium* strains. A previous report showed that IFN‐γ induces splenic inflammatory monocytes (CD11c^+^Ly6C^+^MHCII^hi^DC‐SIGN^hi^) to a population of MO‐DCs (expressing high levels of CCR5, CXCL9, and CXCL10); the CCR5^+^CXCL9/10^+^ MO‐DCs traffic to the brain and amplify an influx of CD8^+^ T cells, leading to a lethal neuro‐pathological syndrome after a *P. b*. ANKA infection.^[^ ^68^ ^]^ Moreover, a coinstantaneous report showed that early type‐I IFN results in a high frequency of CD14^+^Ly6C^+^ monocytes and NK cells after a *P. y*. YM infection.^[^ ^17^ ^]^ Our data suggest that CD11b^+^Ly6C^hi^ proinflammatory monocytes induced by late IL‐6 are detrimental to generating protective immunity against *P. y*. YM and *P. y*. N67C infections as they suppress T cell function. This indicates a universally applicable mechanism by which late IL‐6 and CD11b^+^Ly6C^hi^ proinflammatory monocytes suppress host immunity to multiple strains of *Plasmodium* infections.

Proinflammatory cytokines may play a crucial role in malaria infections and parasite clearance. The early production of proinflammatory cytokines, such as tumor necrosis factor alpha (TNF‐α), IL‐12, and IFN‐γ, may protect the host from severe complications.^[^ ^69^, ^70^ ^]^ However, dysregulated pro‐ and anti‐inflammatory responses may in turn contribute to severe outcomes.^[^ ^71^, ^72^, ^73^ ^]^ A clinical study that used plasma from patients with malaria showed that IFN‐γ, IL‐2, IL‐5, IL‐6, and IL‐12 are significantly increased in patients with mild malaria, while transforming growth factor‐beta (TGF‐β), TNF‐α, IL‐10, and IL‐1β are significantly elevated in patients with cerebral malaria.^[^ ^74^ ^]^ The recent studies on patients with malaria have demonstrated a correlation between elevated levels of inflammatory cytokines and inflammatory intermediate monocytes (CD14^+^CD16^+^).^[^ ^75^, ^76^ ^]^ It has been shown that *P. vivax*‐infected patients exhibit high level of proinflammatory cytokines and high frequencies of CD14^+^ monocytes. An analysis of the expression profiles of cytokines from sorted monocytes indicated that classical (CD14^+^CD16^–^) and intermediate inflammatory (CD14^+^CD16^+^) monocytes, both of which resemble the Ly6C^hi^ monocytes in mice,^[^ ^77^ ^]^ contribute to cytokine production during an acute *P. vivax* infection. Furthermore, CD14^+^CD16^+^ monocytes are the most powerful phagocytes of *P. vivax*‐infected reticulocytes.^[^ ^75^ ^]^ Infectious agents and pathogens other than malaria (such as SARS‐CoV and SARS‐CoV‐2) also develop suppressive responses that increase disease severity and cause host death due to abnormal cytokines. Respiratory syncytial virus (RSV) infections are accompanied by elevated amounts of various cytokines and chemokines, including IL‐6, in both humans and experimentally infected mice.^[^ ^78^, ^79^ ^]^ The depletion of IL‐6 during experimental RSV infections protects mice from the severe disease by increasing IFN‐γ‐secreting virus‐specific T cells in the lungs and airways and reducing the induction of the immune‐regulatory cytokines IL‐10 and IL‐27.^[^ ^80^ ^]^ A study in the pneumonia virus (PVM) infection model showed that *Il6^–/–^* mice are less sensitive compared to WT mice. Further results show that a treatment of immunobiotic *Lactobacillus plantarum* in the respiratory tract contributes to reduce IL‐6 production, thus prolonging mice survival.^[^ ^81^ ^]^ Moreover, upon an infection with uropathogenic *E. coli*, highly expressed IL‐6 resulted in an accumulation of Ly6C^+^ monocytes in the urinary bladder. The administration of soluble gp130 to inhibit IL‐6 trans‐signaling significantly reduces accumulation of Ly6C^+^ monocytes.^[^ ^82^ ^]^ Recent studies also showed that SARS‐CoV‐2 infections induce the overproduction of proinflammatory cytokines, such as IL‐6 and TNF‐α. Treatments to block IL‐6 and IL‐6 receptors (such as tocilizumab, chloroquine, and myo‐inositol) have certain impacts on reducing mortality.^[^ ^83^, ^84^, ^85^, ^86^, ^87^ ^]^ Additionally, a recent study indicated that SARS‐CoV‐2 infections curate cGAS‐STING‐mediated NF‐κB to enrich proinflammatory cytokines.^[^ ^88^ ^]^ Our data consistently showed that cGAS‐STING‐mediated late IL‐6 induces CD11b^+^Ly6C^hi^ proinflammatory monocytes and is pathogenic for host immune response against malaria. Considering our findings and the above data, immunotherapies targeting dysregulated cytokines or suppressive cells may be beneficial when promptly deployed in patients with SARS, malaria and other infectious diseases.

In summary, our study showed a distinct cytokine‐production pattern and host resistance when WT mice, cGAS/STING‐deficient mice and MDA5/MAVS‐deficient mice were challenged with lethal *Plasmodium* N67C and YM infections. However, only the cGAS/STING‐deficient mice were resistant to both the YM and N67C infections. The robust production of type‐I IFN in pDCs in the early stages of YM and N67C infections, as well as the downregulation of IL‐6 production in macrophages in the late stages of N67C infections, produce potent anti‐malaria host immune responses against YM and N67C infections. Specifically, cGAS and STING interact with MyD88 and drive a p38‐mediated IL‐6 production in macrophages, which induces CD11b^+^Ly6C^hi^ proinflammatory monocytes to inhibit the proliferation and function of T cells as well as anti‐malaria host immune responses against N67C infections. Collectively, these findings provide insights into the mechanisms by which cGAS‐STING‐induced IL‐6 controls anti‐malaria immunity and therefore provide potential therapeutic targets for the development of effective vaccines against malaria and, perhaps, other infectious diseases.

## 4 Experimental Section

### Microbes

*Plasmodium yoelii* YM and N67C were original from MR4. Parasite‐infected mice blood was collected in saline solution and filtered to deplete white blood cells. Parasites were spun down after RBC lysis buffer treatment, and lysate incubated with buffer A (150 mm NaCl, 25 mm EDTA, 10% SDS, and protein kinase) overnight. gDNAs were isolated using phenol/chloroform, and RNAs were isolated using TRIzol reagent (Invitrogen).

### Mice

Female mice of C57BL/6 (WT), *Ifih1^–/–^*, *Mavs^–/–^*, *Myd88^–/–^*, *Tmem173^gt^, Il6^–/–^, Mapk14^fl/f^* and *Lyz2‐cre* mice were purchased from The Jackson Laboratory. *Tlr7^–/–^* mice were kindly gifted from Dr. Richard A. Flavell (Yale University). *Tlr9^–/‐^* mice were from Dr. Marco Colonna (Washington University School of Medicine). *Mb21d1^–/–^* mice were from Dr. Skip Virgin (Washington University at St. Louis). Mouse‐related procedures were performed according to experimental protocol (AUP‐0615‐0047) approved by the IACUC at Houston Methodist Research Institute and protocol (21098) approved by the IACUC at University of Southern California. The IACUC uses the NIH *Guide for the Care and Use of Laboratory Animals* (National Academies Press, 2011), which is based on *US Government Principles for Utilization and Care of Vertebrate Animals Used in Testing, Research, and Training* (National Academies Press, 2011). Animal experiments (SMUL2020125) performed in Southern Medical University were approved by the IACUC of Southern Medical University.

### In Vivo Procedures

For *plasmodium* infection, 1×10^6^ iRBCs (otherwise, indicated specifically in the figure legend) suspended in 200 µL PBS from the donor mice were intraperitoneally injected into experimental mice. Recombinant mouse IFN‐α/β (800 U/g) or IL‐6 (50 ng g^−1^) were injected intravenously into WT or deficient mice at indicated time points. To block IL‐6 receptor, anti‐mouse‐IL6 receptor antibodies (500 µg) were injected at indicated time points. To block PD‐1 signaling, anti‐PD‐1 antibodies (Bio X Cell) were injected intraperitoneally at 250 µg per injection at day 2 p.i. and then twice injections a week.

### In Vivo Depletion of Cells

To deplete pDCs, pDC‐depleting functional‐grade mAb (anti‐mPDCA‐1 IgG, clone JF05‐1C2.4.1) and the corresponding isotype control IgG control were purchased from Miltenyi Biotec (Auburn, CA), and two intraperitoneal injections of antibody (250 µg/mouse) were administered 12 h prior and after the indicated time points. To deplete macrophages, clodronate liposomes (from Dr. Nico. Van Rooijen) were injected intraperitoneally at 750 µg per injection at the indicated time points, and control liposomes served as control. To deplete proinflammatory monocytes, anti‐Ly6C antibody (25 µg g^−1^) (Bio X Cell) was injected intraperitoneally at indicated time points, and rat IgG served as control.

### Cell Culture

RAW264.7 cells were cultured in RPMI1640 medium (Gibco) with 10% FBS (Gibco) and 1% penicillin‐streptomycin (Gibco). HEK293T (human embryonic kidney 293T) cells and L929 cells were cultured in DMEM (Gibco) supplemented with 10% FBS and 1% penicillin‐streptomycin. The method to produce L929 conditioned medium from Virginie et al. was followed.^[^ ^89^ ^]^

### Primary Cells Isolation

Bone marrow cells were isolated from the tibia and femur and cultured in RPMI1640 medium with 10% FBS, 1% penicillin‐streptomycin, 55 µm β‐mercaptoethanol and 10% L929 conditioned media containing macrophage‐colony stimulating factor (M‐CSF) for 6 days to harvest BMDMs. Mouse T cells were isolated from spleens by using the Dynabeads Untouched Mouse T Cells Kit (Thermo Fisher). CD11b^+^Ly6C^hi^Ly6G^–^ cells were sorted on a MoFlo Astrios instrument. For CD11b^+^Ly6G^+^ isolation, cells were labeled with biotinylated anti‐Ly6G (BioLegend), incubated with streptavidin microbeads (BD), and separated on magnetic columns (Stemcell). For specific cell isolation from splenocytes, pDCs were isolated using anti‐mPDCA‐1 microbeads from Miltenyi Biotec (Auburn, CA). After pDCs isolation, macrophages were isolated with CD11b microbeads from Miltenyi Biotec, cDCs were isolated with mouse CD11c PE labeling and followed by PE selection cocktail from STEMCELL technologies, following the manufacturer's protocol. RNAs from pDCs, cDCs, and macrophages were isolated using TRIzol reagent (Invitrogen) and subjected to semi‐quantitative PCR analysis of 18S rRNA by using specific primer.

### Immunoprecipitation and Immunoblot Analyses

For immunoprecipitation, whole‐cell lysates were incubated overnight with indicated antibodies plus protein A and G beads (Pierce). For immunoprecipitation with anti‐FLAG, anti‐FLAG agarose gels (BioLegend) were used. Beads were then washed 5 times with low‐salt lysis buffer, and immunoprecipitates were eluted with 4× SDS loading buffer. Immunoblotting was performed by resolving protein lysates on SDS‐PAGE gels, followed by transfer to PVDF membranes (Bio‐Rad) and further incubation of membranes with indicated antibodies overnight. For all blots, EMD Millipore Luminata Western HRP Chemiluminescence substrate was used for protein detection. Anti‐Flag antibody was purchased from Sigma‐Aldrich; anti‐HA antibody was purchased from Roche; anti‐STING, anti‐cGAS, anti‐p‐p38, anti‐p38, anti‐p‐p65, anti‐p‐ERK, anti‐p‐ERK, anti‐JNK, and anti‐p‐JNK antibodies were purchased from Cell Signaling Technology; anti‐MyD88 antibody and anti‐β‐actin antibody were purchased from Santa Cruz Biotechnology.

### Flow Cytometry

Single‐cell suspensions from spleen were obtained from tissues and stained for 20 min with indicated antibodies. For intracellular staining, T cells were stimulated with eBioscience Cell Stimulation Cocktail for 6 h at 37 °C in the presence of GolgiStop. Cells were then stained with the surface marker for 15 min on ice and permeabilized using Cytofix/Cytoperm for 30 min on ice. Permeabilized cells were resuspended in Perm/Wash buffer and stained with cytokine antibody for 20 min. FACS analysis was performed with BD LSRII Flow Cytometer (BD) or Attune Flow Cytometers (ThermoFisher Scientific), and data were analyzed by BD FACSDiva, Attune NxT Software, or Flowjo software. For specific T cell analysis, splenocytes were harvested and stained with anti‐CD4, anti‐CD11a, and anti‐CD49d antibodies. Anti‐Foxp3, anti‐CD4, anti‐CD3, anti‐CD25, anti‐CD8, anti‐IFN‐γ, anti‐CD11b, anti‐Gr1, anti‐Ly6C, anti‐Ly6G, anti‐CD11a, and anti‐CD49d antibodies were purchased from BioLegend, eBioscience or Invitrogen.

### ELISA

Mouse sera or cell supernatants were collected at the indicated time after infection or stimulation and subjected to analysis with commercial ELISA kits for mouse IFN‐α, IFN‐β, (PBL Biomedical Laboratories) or IFN‐γ, IL‐6 (eBioscience), following the manufacturer's instructions.

### Immunohistochemistry

Fresh spleens were fixed with 3.7% formalin for 24 h, and then sent to the histology core at Baylor Breast Care Center for further processing and H&E staining. IHC staining was performed with the streptavidin‐biotin‐peroxidase complex method using the Vectastain Elite ABC Kit and the DAB Peroxidase (HRP) Substrate Kit (Vector Laboratories). Anti‐CD3 (ab16669), Goat anti‐Rabbit IgG (A11034), and ProLong Gold Antifade Mountant with DAPI (P36931) were purchased from Abcam or ThermoFisher. Negative control staining was performed using mouse, rabbit, or rat immune sera instead of the primary antibodies.

### T Cell Proliferation Assay

CD3^+^ T cells were isolated from spleen with biotin‐anti‐mouse CD3ε antibody plus strpv IMag particles, and labeled with CFSE (2 µM), then stimulated with soluble anti‐CD3 and anti‐CD28, cultured alone or with isolated Ly6C^hi^ or Ly6G^hi^ cells from N67C infected mice at different ratios for 3 days. Cells were then stained with anti‐CD4 and anti‐CD8 antibodies, and T cell proliferation was analyzed by flow cytometry.

### RNA Preparation and qPCR

Total RNAs were harvested from splenic tissues, lymph nodes, or stimulated cells using the TRIzol reagent (Invitrogen), and the complimentary cDNAs were generated using reverse transcriptase IV (Invitrogen). Real‐time PCR was performed using the ABI Q6 analyzer (Applied Biosystems) and using iTaq SYBR Green Supermix (BioRad) with specific primers.

### Statistical Analysis

All analyses were performed using GraphPad Prism version 5.0 (GraphPad Software, La Jolla, CA). Data are presented as mean ± s.d., unless otherwise stated. Differences in mice survival were evaluated with Mantel–Cox log‐rank test. The sample size for each experiment, *n*, is included in the results section and the associated figure legend. Statistical significance of differences between two groups was assessed by unpaired Student *t* tests and a *p* value of <0.05 was considered significant.

## Conflict of Interest

The authors declare no conflict of interest.

## Author Contributions

Y.D., Y.L., and Z.H., contributed equally to this project. Y.D., X.Y., Y.L., Z.H., and J.L., designed and performed the experiments. X.L., C.X., T.D., and J.C. provided assistance or technique support in some experiments. J.W. and X.‐z.S. provided malaria strains and some experimental assistance. H.Y.W., Y.D., X.‐z.S., X.Y., and R.‐F.W. performed data analysis and wrote the manuscript. R.‐F.W supervised the entire project.

## Supporting information

Supporting Information

## Data Availability

The data that support the findings of this study are available from the corresponding author upon reasonable request.
