# Supplementary material for: Activation of cGAS‐STING by Lethal Malaria N67C Dictates Immunity and Mortality through Induction of CD11b+Ly6Chi Proinflammatory Monocytes
Source: Adv Sci (Weinh). 2022 May 29;9(22):2103701. doi: 10.1002/advs.202103701 (PMC9353503; doi:10.1002/advs.202103701)
Supplement: Supplementary file 1 — Supporting Information [file ADVS-9-2103701-s001.pdf]

Supporting Information

Activation of cGAS-STING by Lethal Malaria N67C Dictates Immunity and Mortality through Induction of $CD11b^{+}Ly6C^{hi}$ Proinflammatory Monocytes

Yang Du, Yien Luo, Zhiqiang Hu, Jiansen Lu, Xin Liu, Changsheng Xing, Jian Wu, Tianhao Duan, Junjun Chu, Helen Y. Wang, Xin-zhuan Su, Xiao Yu, and Rong-Fu Wang**

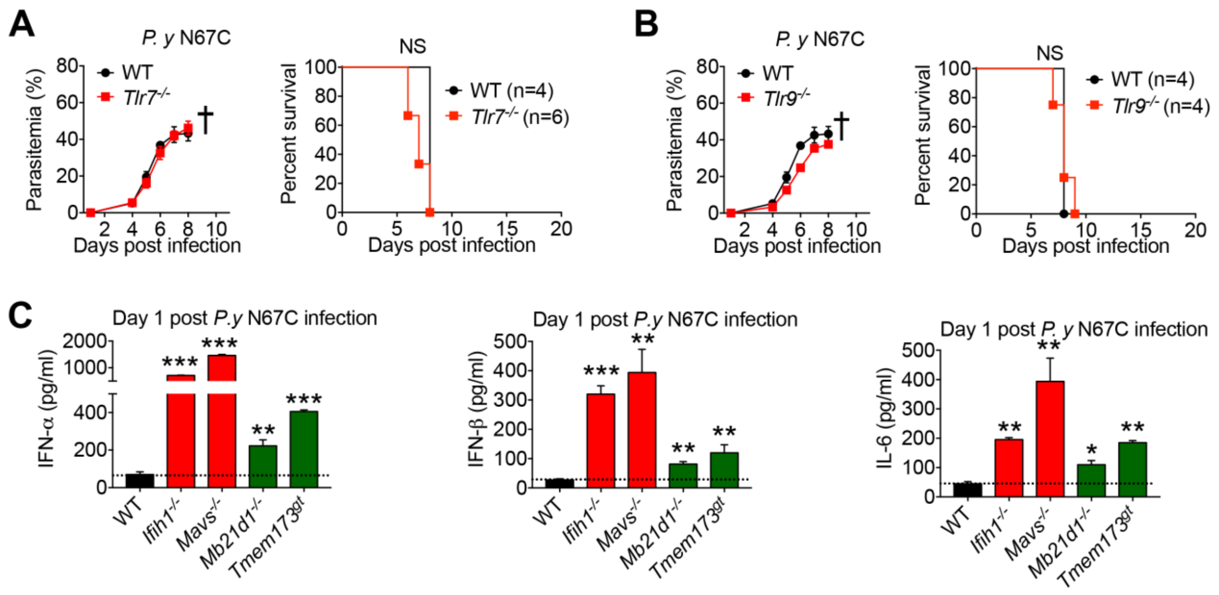

Figure S1. TLR related genes deficient mice are sensitive to N67C infection.

A, B) WT (black lines), *Tlr7*$^{-/-}$ A) and *Tlr9*$^{-/-}$ B) mice were intraperitoneally infected with N67C (1×10^6 iRBCs). Daily parasitemia and mortality rates are shown. C) WT, *Ifih1*$^{-/-}$, *Mavs*$^{-/-}$, *Mb21d1*$^{-/-}$ and *Tmem173*^{gt} mice (n=5) were intraperitoneally infected with N67C. Sera were collected at day 1 *p.i.* and subjected to ELISA analysis of IFN-α, IFN-β and IL-6. Data are representative of three independent experiments and are plotted as the mean ±SD. **p*<0.05, ***p*<0.01, ****p*<0.001 vs. corresponding control. NS, not significant. † denotes mouse death.

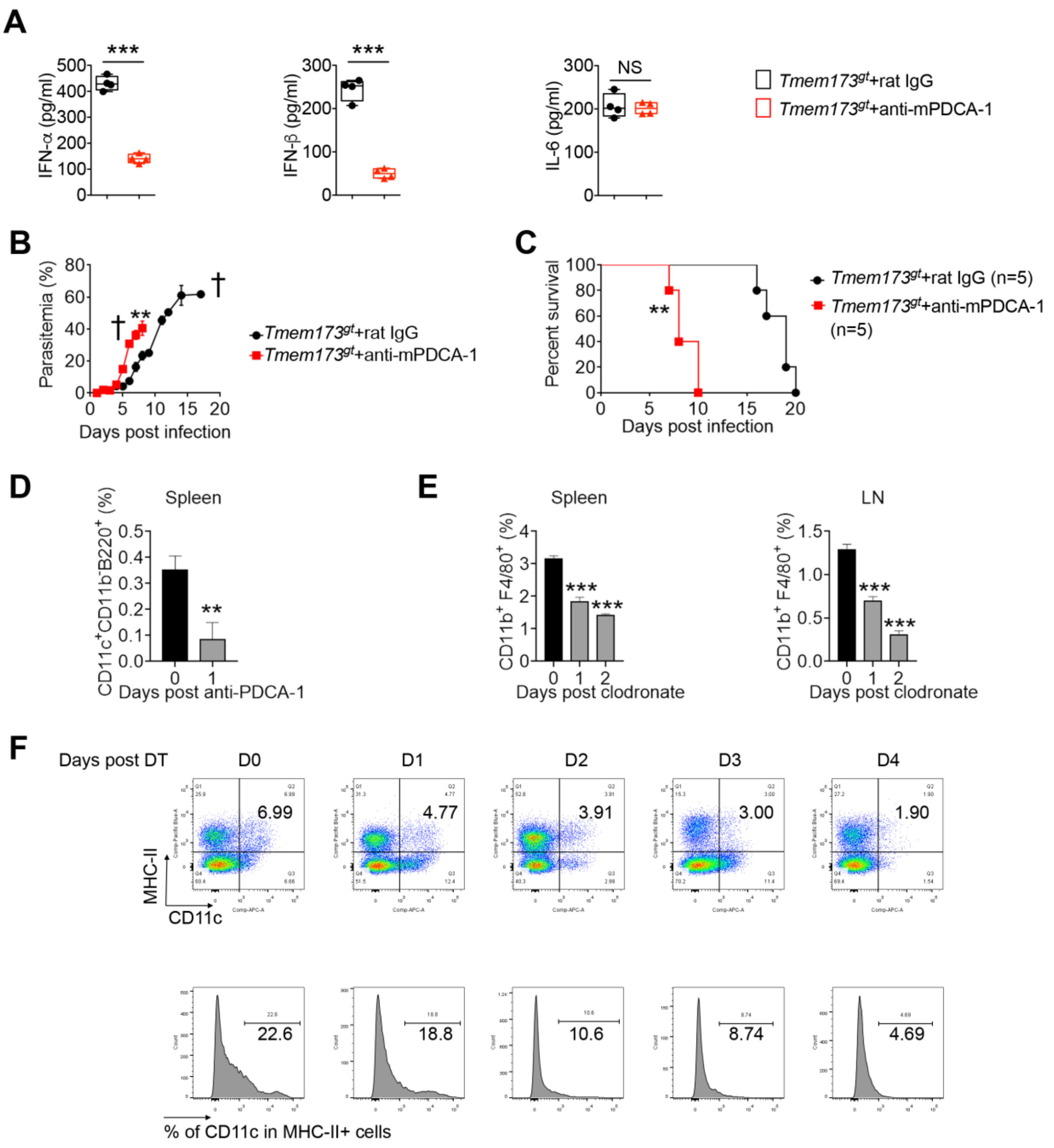

Figure S2. pDCs contribute to the production of early type I IFN and prolong host survival.

A-C) *Tmem173^{gt}* mice (n=5) were infected with N67C, followed by anti-m-PDCA-1 antibody injection at 12 h before and after infection. Sera were collected at day 1 *p.i.* and subjected to ELISA analysis of IFN-α, IFN-β and IL-6 A). Parasitemia B) and mortality rates C) were monitored daily. D) Depletion efficiency of pDCs after anti-PDCA-1 injection. WT mice (n=3) were injected with anti-PDCA-1 antibodies (250 μg/mouse). Cells from spleens were collected at

indicated time points and subjected to FACS analysis. E) Depletion efficiency of macrophages after clodronate injection. WT mice (n=3) were injected with clodronate (700 mg, intraperitoneally). Cells from spleens and lymph nodes were collected at indicated time points and subjected to FACS analysis. F) Depletion efficiency of cDCs after DT injection. zDC-DTR>WT chimeric mice were administrated with DT (100 ng), spleens were harvested at indicated time points post DT injection and subjected to FACS analysis of cDCs. Data are representative of three independent experiments and are plotted as the mean ±SD. $^{**}p<0.01$, $^{***}p<0.001$ vs. corresponding control. NS, not significant. **†** denotes mouse death.

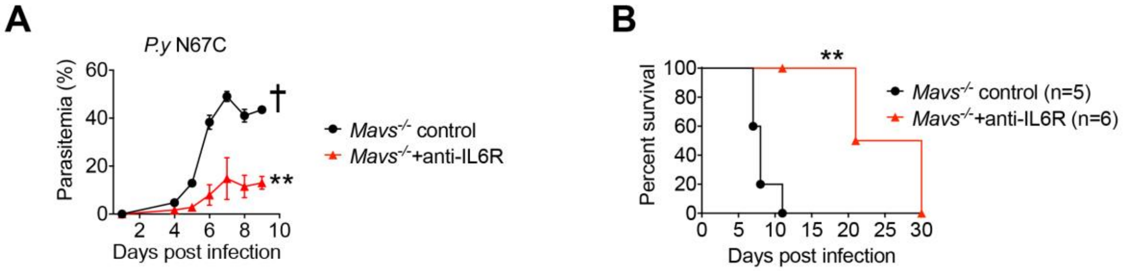

Figure S3. IL-6 plays a detrimental role in generating immunity against N67C infection.

A, B) *Mavs*$^{-/-}$ mice (n=5) were infected with N67C, followed by blockage of IL-6R with anti-IL6R antibody (500 μg) at day 3 post N67C infection, parasitemia A) and mortality rates B) were monitored daily. Data are representative of three independent experiments and are plotted as the mean ±SD. $^{**}p<0.01$ vs. corresponding control. † denotes mouse death.

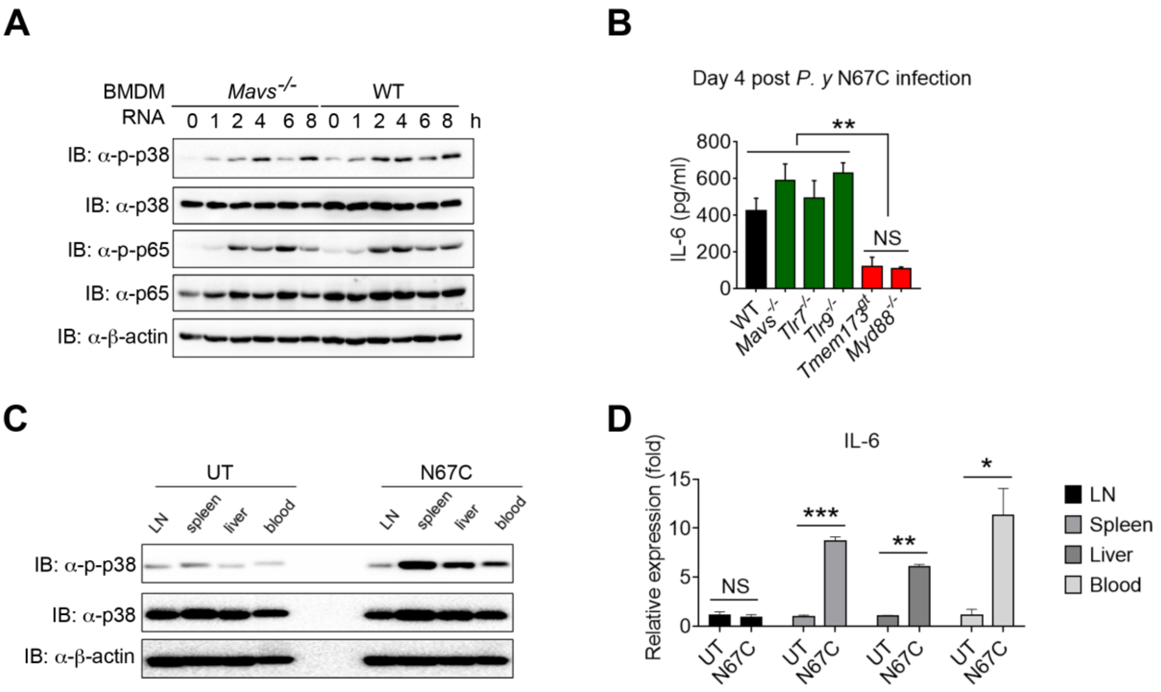

Figure S4. MAVS and TLRs are not involved in *Plasmodium* induced IL-6 production.

A) WT and *Mavs*$^{-/-}$ BMDMs were stimulated with RNA for indicated time points, cell lysates were analyzed by immunoblotting with the indicated antibodies. B) WT, *Mavs*$^{-/-}$, *Tlr7*$^{-/-}$, *Tlr9*$^{-/-}$, *Myd88*$^{-/-}$and *Tmem173*^{gt} mice were intraperitoneally infected with N67C. Sera were collected at day 4 *p.i.* and subjected to ELISA analysis of IL-6 (n=3). C, D) *Mavs*$^{-/-}$ mice were injected with N67C. Cells from lymph node, spleen, liver and blood were collected at day 3 *p.i.* and subjected to immunoblot analysis C) and expression analysis using qPCR (n=3) D). Data are representative of three independent experiments and are plotted as the mean ±SD. $^{*}p<0.05$, $^{**}p<0.01$, $^{***}p<0.001$ vs. corresponding control. NS, not significant.

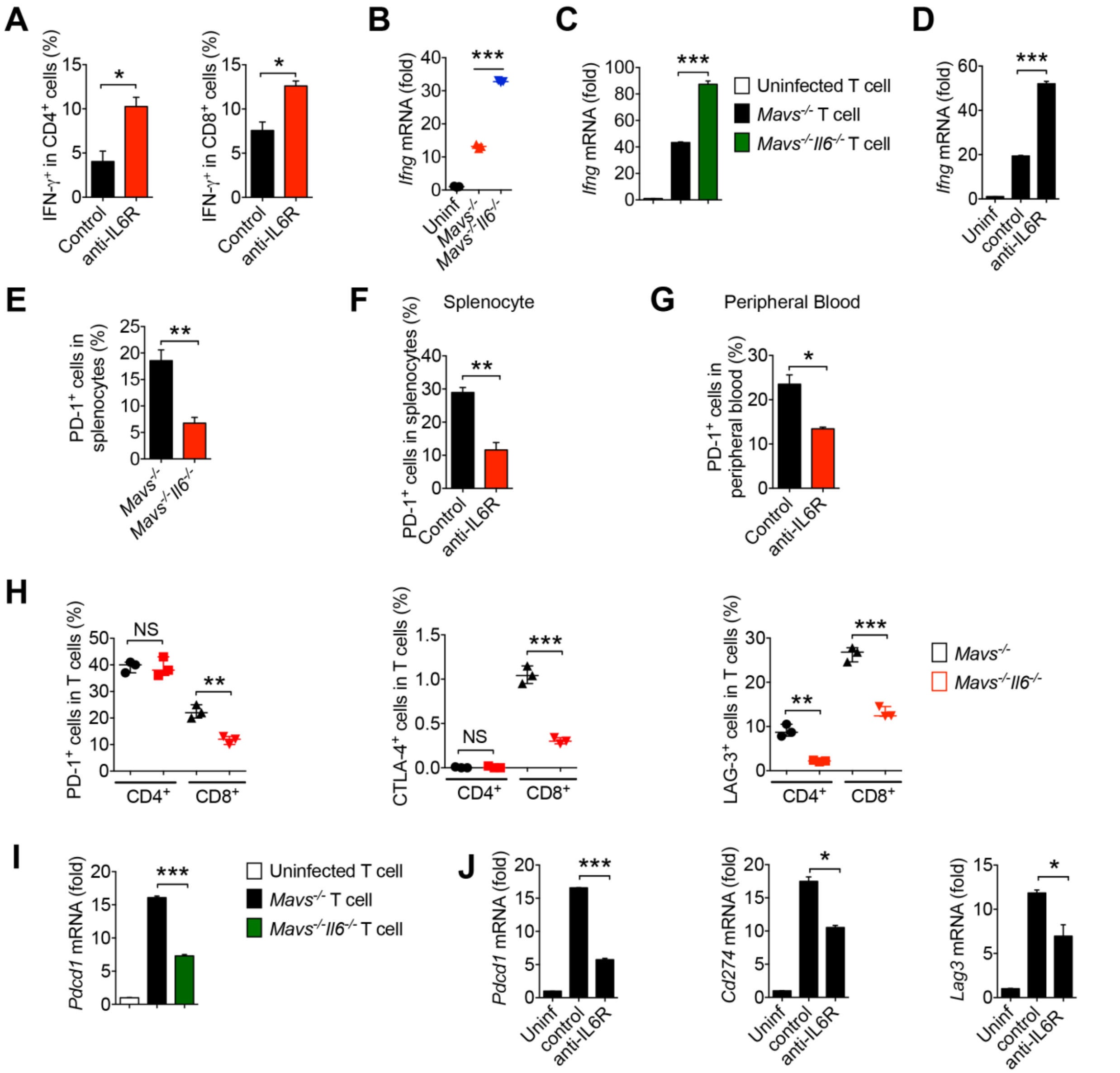

Figure S5. Blockage of IL-6 signaling promotes host adaptive immune responses against N67C infection by enhancing T cell function.

A) *Mavs*$^{-/-}$ mice were infected with N67C, and then treated with control or anti-IL6R antibody at day 2 *p.i.*. Splenocytes were collected at day 5 *p.i.* and stimulated with N67C crude antigen *in vitro*, then subjected to FACS analysis of IFN-γ^{+} cells in CD4^{+} cells and in CD8^{+} cells. B) *Mavs*$^{-/-}$ and *Mavs*$^{-/-}$*Il6*$^{-/-}$ mice were infected with N67C. Splenocytes were collected at day 5 *p.i.*. RNAs from splenocytes were isolated and used for expression analysis by using qPCR. C) *Mavs*$^{-/-}$ and

Mavs$^{-/-}$*Il6*$^{-/-}$ mice were infected with N67C. Splenocytes were collected at day 5 *p.i.*. T cells were isolated from splenocytes with specific untouched beads and RNAs from T cells were isolated and used for expression analysis by using qPCR. D) *Mavs*$^{-/-}$ mice were infected with N67C, and then treated with control or anti-IL6R antibody at day 2 *p.i.*, splenocytes were collected at day 5 *p.i.*. RNAs from splenocytes were isolated and used for expression analysis by using qPCR. E) *Mavs*$^{-/-}$ and *Mavs*$^{-/-}$*Il6*$^{-/-}$ mice were infected with N67C. Splenocytes were collected at day 5 *p.i.*, and subjected to FACS analysis of PD-1^{+} cells in splenocytes. F, G) *Mavs*$^{-/-}$ mice were infected with N67C, and then treated with or without anti-IL6R antibody at day 2 *p.i.*. Splenocytes F) and peripheral blood G) were collected at day 5 *p.i.*, and subjected to FACS analysis of PD-1^{+} cells. H) *Mavs*$^{-/-}$ and *Mavs*$^{-/-}$*Il6*$^{-/-}$ mice were infected with N67C, splenocytes were collected at day 5 *p.i.*, and subjected to FACS analysis of PD-1^{+} cells, CTLA-4^{+} cells and LAG-3^{+} cells in CD4^{+} T cells and CD8^{+} T cells. I) *Mavs*$^{-/-}$ and *Mavs*$^{-/-}$*Il6*$^{-/-}$ mice were infected with N67C. Splenocytes were collected at day 5 *p.i.*. T cells were isolated from splenocytes with specific untouched beads and RNAs from T cells were isolated and used for expression analysis by using qPCR. J) *Mavs*$^{-/-}$ mice were infected with N67C, and then treated with or without anti-IL6R antibody at day 2 *p.i.* Splenocytes were collected at day 5 *p.i.*. RNAs from splenocytes were isolated and used for expression analysis by using qPCR. Data are representative of three independent experiments and are plotted as the mean ±SD. $^{*}p<0.05$, $^{**}p<0.01$, $^{***}p<0.001$ vs. corresponding control. NS, not significant. (A-J, n=3).

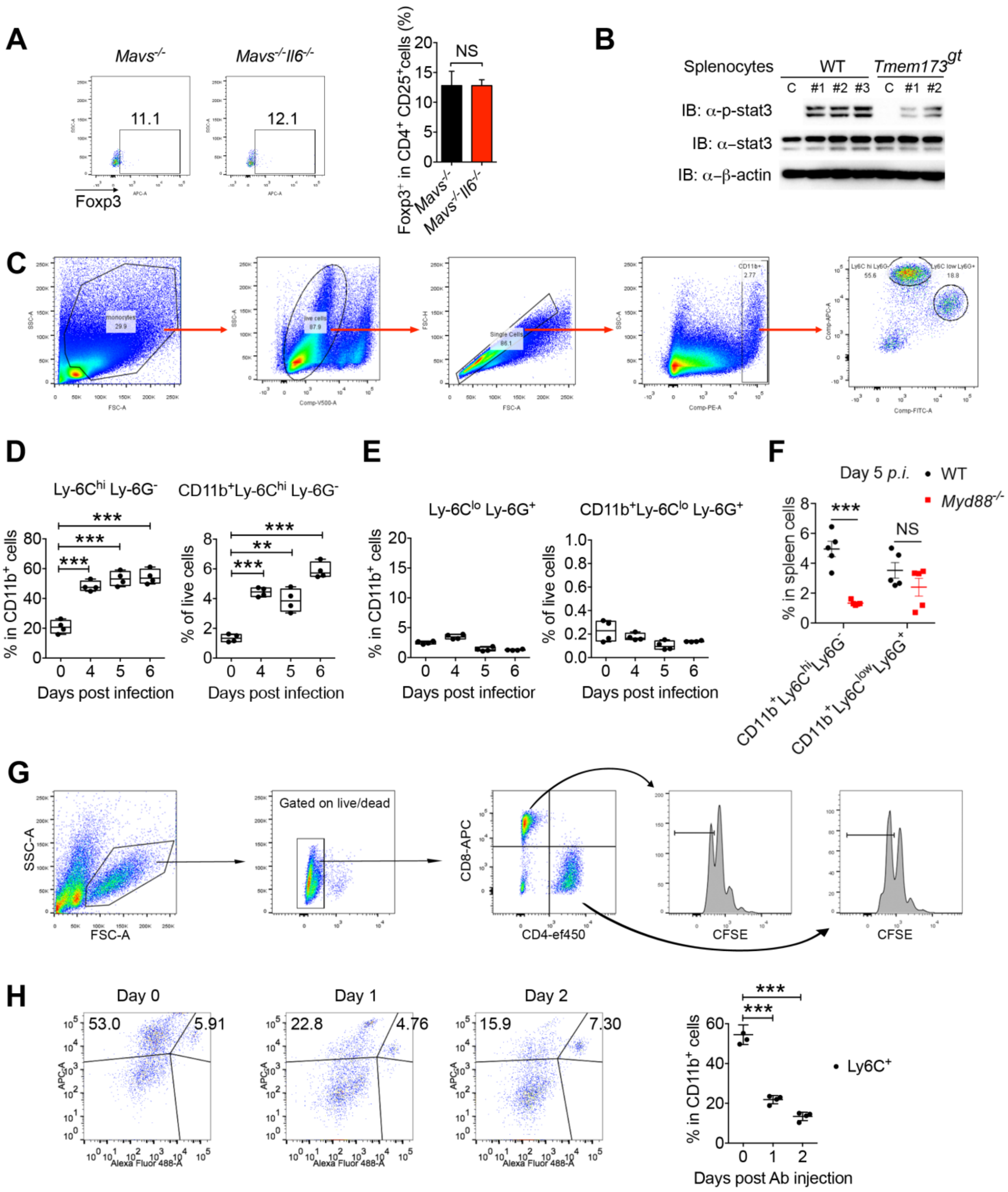
A
Mavs-/-
Mavs-/-Il6-/-
11.1
12.1
Foxp3
Foxp3+ in CD4+ CD25+cells (%)
NS
Mavs-/-
Mavs-/-Il6-/-
B
Splenocytes
WT
Tmem173gt
C #1 #2 #3 C #1 #2
IB: α-p-stat3
IB: α-stat3
IB: α-β-actin
C
D
Ly-6Chi Ly-6G-
CD11b+Ly-6Chi Ly-6G-
% in CD11b+ cells
% of live cells
Days post infection
E
Ly-6Clo Ly-6G+
CD11b+Ly-6Clo Ly-6G+
F
Day 5 p.i.
WT
Myd88-/-
% in spleen cells
NS
CD11b+Ly6ChiLy6G-
CD11b+Ly6ClowLy6G+
G
SSC-A
FSC-A
Gated on live/dead
CD8-APC
CD4-ef450
CFSE
H
Day 0
Day 1
Day 2
53.0
5.91
22.8
4.76
15.9
7.30
APC-A
Alexa Fluor 488-A
% in CD11b+ cells
Ly6C+
Days post Ab injection

Figure S6. IL-6 signaling inhibits T cell function by inducing $CD11b^{+}Ly6C^{hi}$ proinflammatory monocytes expansion.

A) *Mavs*$^{-/-}$ and *Mavs*$^{-/-}$*Il6*$^{-/-}$ mice (n=2) were infected with N67C. Splenocytes were collected at day 5 *p.i.*, and subjected to FACS analysis of $Foxp3^{+}$ cells in $CD4^{+}CD25^{+}$cells. B) WT and *Tmem173*^{gt} mice were infected with N67C. Splenocytes were collected at day 3.5 *p.i.*, and cell lysates were analyzed by immunoblotting with the indicated antibodies. C) FACS gating strategy for $Ly6C^{hi}Ly6G^{-}$ cells and $Ly6C^{lo}Ly6G^{+}$ cells. D, E) WT mice (n=4) were infected with N67C. Splenocytes were collected at the indicated time points and subjected to FACS analysis of $Ly6C^{hi}Ly6G^{-}$ cells D) and $Ly6C^{lo}Ly6G^{+}$ cells E) in $CD11b^{+}$ cells and live cells. F) WT and *Myd88*$^{-/-}$ mice (n=5) were infected with N67C. Splenocytes were collected at day 5 *p.i.*, and subjected to FACS analysis of $CD11b^{+}Ly6C^{hi}Ly6G^{-}$ cells and $CD11b^{+}Ly6C^{lo}Ly6G^{+}$ cells. G) FACS gating strategy for CFSE-labeled T cell proliferation assay. H) Depletion efficiency of $Ly6C^{+}$ cells after anti-Ly6C antibody treatment (n=4). Data are representative of three independent experiments and are plotted as the mean ±SD. $^{*}p<0.05$, $^{**}p<0.01$, $^{***}p<0.001$ vs. corresponding control. NS, not significant.

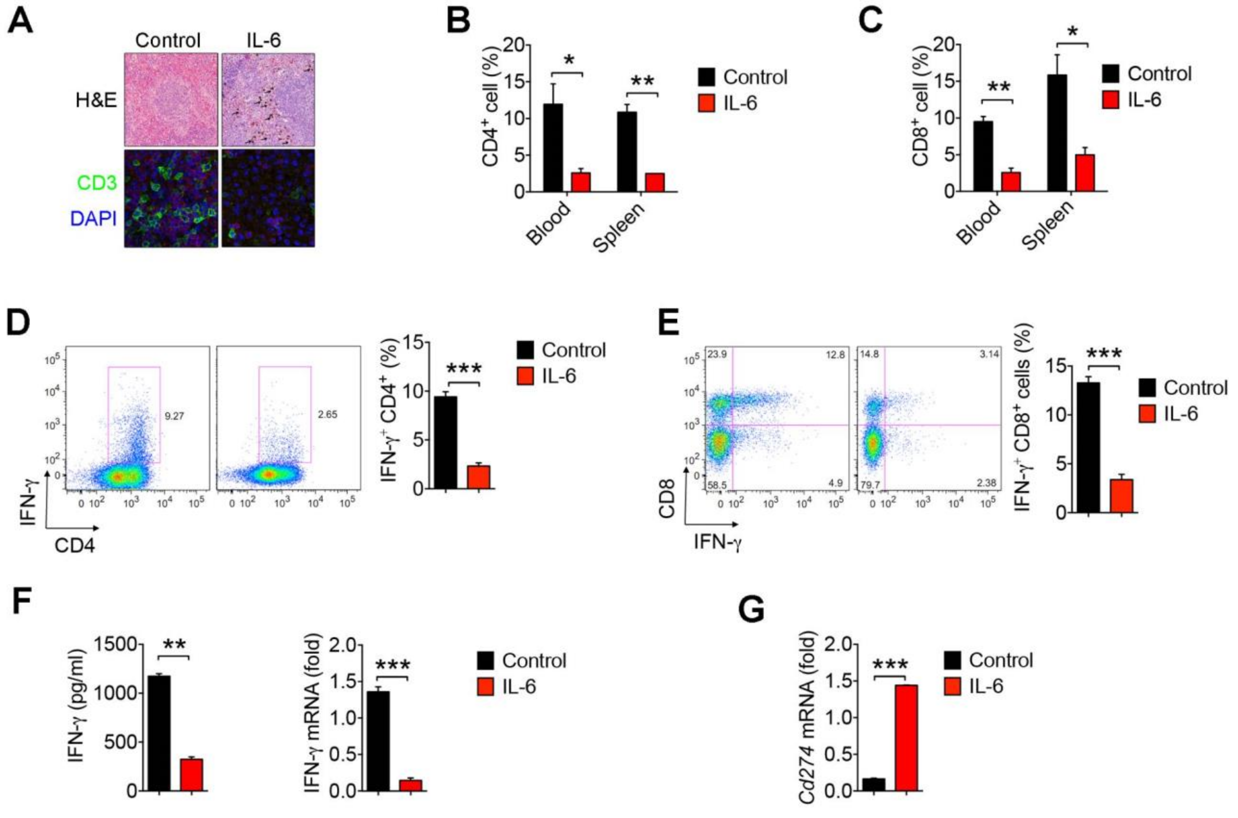

Figure S7. Administration of IL-6 is detrimental for host generating immune responses against lethal malaria infection.

A-C) *Mavs*$^{-/-}$ mice (n=3) were infected with YM, and then treated with or without recombinant IL-6 at day 3 *p.i.*. H&E staining (10×) and IF staining of CD3 (40×) on spleen sections collected at day 5 were shown in A). Peripheral blood and splenocytes were collected at day 5 *p.i.*, and subjected to FACS analysis of CD4^{+} cells B) and CD8^{+} cells C). D, E) *Mavs*$^{-/-}$ mice (n=3) were infected with YM, and then treated with or without recombinant IL-6 at day 3 *p.i.*. Splenocytes were collected at day 5 *p.i.*, and subjected to FACS analysis of IFN-γ^{+} cells in CD4^{+} cells D) and CD8^{+} cells E). F, G) *Mavs*$^{-/-}$ mice (n=3) were infected with YM, and then treated with or without recombinant IL-6 at day 3 *p.i.*. Sera were collected at day 5 *p.i.* and subjected to ELISA analysis of IFN-γ F). Splenocytes were collected at day 5 *p.i.*. RNAs from splenocytes were isolated and used for expression analysis by using qPCR F and G). Data are representative of three independent experiments and are plotted as the mean ±SD. $^{*}p<0.05$, $^{**}p<0.01$, $^{***}p<0.001$ vs. corresponding control.

Supplementary Table. Primers sequences for quantitative PCR

**Description gene/protein**	**Forward Primer Sequence**	**Reverse Primer Sequence**
Pdcd1 / PD-1	ACCCTGGTCATTCACTTGGG	CATTTGCTCCCTCTGACACTG
Cd274 / PDL-1	GCTCCAAAGGACTTGTACGTG	TGATCTGAAGGGCAGCATTTC
Cd273 / PDL-2	CTGCCGATACTGAACCTGAGC	GCGGTCAAAATCGCACTCC
Havcr2 / TIM-3	TCAGGTCTTACCCTCAACTGTG	GGGCAGATAGGCATTTTTACCA
Cd223 / LAG-3	CTGGGACTGCTTTGGGAAG	GGTTGATGTTGCCAGATAACCC
Cd152 / CTLA-4	TTTTGTAGCCCTGCTCACTCT	CTGAAGGTTGGGTCACCTGTA
Ifng / IFN-γ	ATGAACGCTACACACTGCATC	CCATCCTTTTGCCAGTTCCTC
Gapdh / GAPDH	AAGGTCATCCCAGAGCTGAA	CTGCTTCACCACCTTCTTGA
18S rRNA	CTTGGCTCCGCCTCGATAT	TCAAAGTAACGAGAGCCCAATG
